# ST6GAL1 sialyltransferase promotes acinar to ductal metaplasia and pancreatic cancer progression

**DOI:** 10.1172/jci.insight.161563

**Published:** 2023-10-09

**Authors:** Nikita Bhalerao, Asmi Chakraborty, Michael P. Marciel, Jihye Hwang, Colleen M. Britain, Austin D. Silva, Isam E. Eltoum, Robert B. Jones, Katie L. Alexander, Lesley E. Smythies, Phillip D. Smith, David K. Crossman, Michael R. Crowley, Boyoung Shin, Laurie E. Harrington, Zhaoqi Yan, Maigen M. Bethea, Chad S. Hunter, Christopher A. Klug, Donald J. Buchsbaum, Susan L. Bellis

**Affiliations:** 1 Department of Cell, Developmental, and Integrative Biology;; 2 Department of Pathology;; 3 Department of Medicine;; 4 Department of Genetics;; 5 Department of Microbiology; and; 6 Department of Radiation Oncology, University of Alabama at Birmingham, Birmingham, Alabama, USA.

**Keywords:** Oncology, Cancer, Glycobiology, Oncogenes

## Abstract

The role of aberrant glycosylation in pancreatic ductal adenocarcinoma (PDAC) remains an under-investigated area of research. In this study, we determined that ST6 β-galactoside α2,6 sialyltransferase 1 (ST6GAL1), which adds α2,6-linked sialic acids to N-glycosylated proteins, was upregulated in patients with early-stage PDAC and was further increased in advanced disease. A tumor-promoting function for ST6GAL1 was elucidated using tumor xenograft experiments with human PDAC cells. Additionally, we developed a genetically engineered mouse (GEM) model with transgenic expression of ST6GAL1 in the pancreas and found that mice with dual expression of ST6GAL1 and oncogenic KRAS^G12D^ had greatly accelerated PDAC progression compared with mice expressing KRAS^G12D^ alone. As ST6GAL1 imparts progenitor-like characteristics, we interrogated ST6GAL1’s role in acinar to ductal metaplasia (ADM), a process that fosters neoplasia by reprogramming acinar cells into ductal, progenitor-like cells. We verified ST6GAL1 promotes ADM using multiple models including the 266-6 cell line, GEM-derived organoids and tissues, and an in vivo model of inflammation-induced ADM. EGFR is a key driver of ADM and is known to be activated by ST6GAL1-mediated sialylation. Importantly, EGFR activation was dramatically increased in acinar cells and organoids from mice with transgenic ST6GAL1 expression. These collective results highlight a glycosylation-dependent mechanism involved in early stages of pancreatic neoplasia.

## Graphical abstract

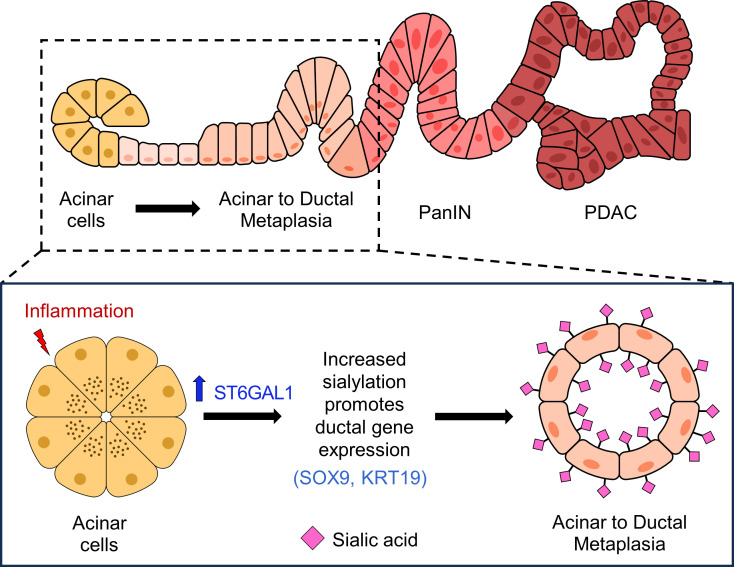

## Introduction

Pancreatic ductal adenocarcinoma (PDAC) is an aggressive cancer with a 5-year survival rate of approximately 10% (1). Accordingly, there is a pressing need to elucidate molecular mechanisms underlying PDAC development. One of the earliest events contributing to pancreatic neoplasia is acinar to ductal metaplasia (ADM), a process in which acinar cells acquire a more ductal, progenitor-like phenotype (2, 3). The disease then progresses through varying stages of premalignant lesions called pancreatic intraepithelial neoplasias (PanINs), followed by transition to adenocarcinoma (4–6). More than 90% of patients with PDAC have activating mutations in *KRAS* (4); however, compared with other cancer types, PDAC has a relatively low mutational load.

In tandem with mutational events, proteins in cancer cells are dysregulated through alterations in posttranslational modifications. Among these modifications, abnormal glycosylation has long been associated with carcinogenesis (7–9). In particular, tumor cells often display elevated surface sialylation as a consequence of increased expression of various Golgi sialyltransferases, including ST6 β-galactoside α2,6 sialyltransferase 1 (ST6GAL1) (10, 11). ST6GAL1 is overexpressed in numerous malignancies, and high ST6GAL1 expression correlates with a poor prognosis (12–14). ST6GAL1 adds an α2,6-linked sialic acid to select N-glycosylated proteins destined for the plasma membrane or secretion. Through the α2,6 sialylation of receptors — such as the β1 integrin (15, 16), the Fas and TNF receptor 1 (TNFR1) death receptors (17, 18), and the receptor tyrosine kinases, epidermal growth factor receptor (EGFR), hepatocyte growth factor receptor (also known as MET), and human epidermal growth factor receptor 2 (HER2/erbB2) (19–24) — ST6GAL1 promotes tumor cell migration and invasion, apoptosis resistance, and epithelial-mesenchymal transition (EMT). ST6GAL1 also confers cancer stem cell (CSC) characteristics (25, 26), which may be due, in part, to its activity in upregulating the expression of transcription factors such as SRY-box transcription factor 9 (SOX9) (25). In nonmalignant tissues, ST6GAL1 expression is enriched in certain stem cell populations and tissue niches (26, 27). Furthermore, ST6GAL1 is upregulated when somatic cells are reprogrammed into induced pluripotent stem cells (iPSCs), and ST6GAL1 knockdown hinders iPSC reprogramming (26, 28, 29). More recently, a role for ST6GAL1 in tissue regeneration has been suggested. Punch et al. reported that ST6GAL1 is essential for regeneration of the gastrointestinal tract following whole-body irradiation of mice, and it was hypothesized ST6GAL1 functions in this context to protect intestinal stem cells (30).

The dedifferentiation of acinar cells into ductal-like, progenitor cells during ADM is crucial for pancreatic regeneration following damage caused by inflammation and other injuries (2, 3, 31). Acinar cells undergoing ADM reenter the cell cycle in order to repair the tissue. ADM-like cells typically revert to a differentiated acinar state upon tissue healing (2); however, if these cells acquire oncogenic *KRAS* mutations, they undergo neoplastic transformation, leading to the generation of PanINs (2). Many studies have identified SOX9 as a critical mediator of ADM. SOX9 is upregulated in ADM-like cells, and genetically engineered mouse (GEM) models have confirmed the importance of SOX9 in the formation of ADM and PanIN lesions (32, 33). In fact, Kopp et al. suggested that SOX9-driven ADM is a key mechanism underlying PDAC initiation (32). SOX9 expression is induced in ADM-like cells through signaling by EGFR (34). EGFR is activated within the inflammatory milieu by its ligand, TGF-α, and both TGF-α and EGFR are potent activators of ADM (35–38).

In view of our prior evidence indicating that ST6GAL1 activates EGFR (via sialylation) (19–22), stimulates SOX9 upregulation (25), and confers progenitor characteristics (25, 26), we investigated whether ST6GAL1 facilitates ADM. We first verified ST6GAL1 overexpression in PDAC patient tissues, then established a tumor driver function for ST6GAL1 using tumor xenograft models and GEM models with dual expression of ST6GAL1 and oncogenic KRAS (KRAS^G12D^). Multiple in vitro and in vivo approaches were subsequently employed to show that ST6GAL1 plays a causal role in promoting ADM. Finally, we observed that acinar cells and organoids from mice with transgenic ST6GAL1 expression had dramatically increased activation of EGFR, highlighting a potential mechanism by which ST6GAL1-mediated sialylation fuels ADM development. In the aggregate, these data suggest that ST6GAL1 may contribute to neoplasia by inducing acinar cells to adopt progenitor-like characteristics.

## Results

### ST6GAL1 is upregulated in PDAC patient tissues and promotes PDAC progression in tumor xenograft models.

**Figure 1:**
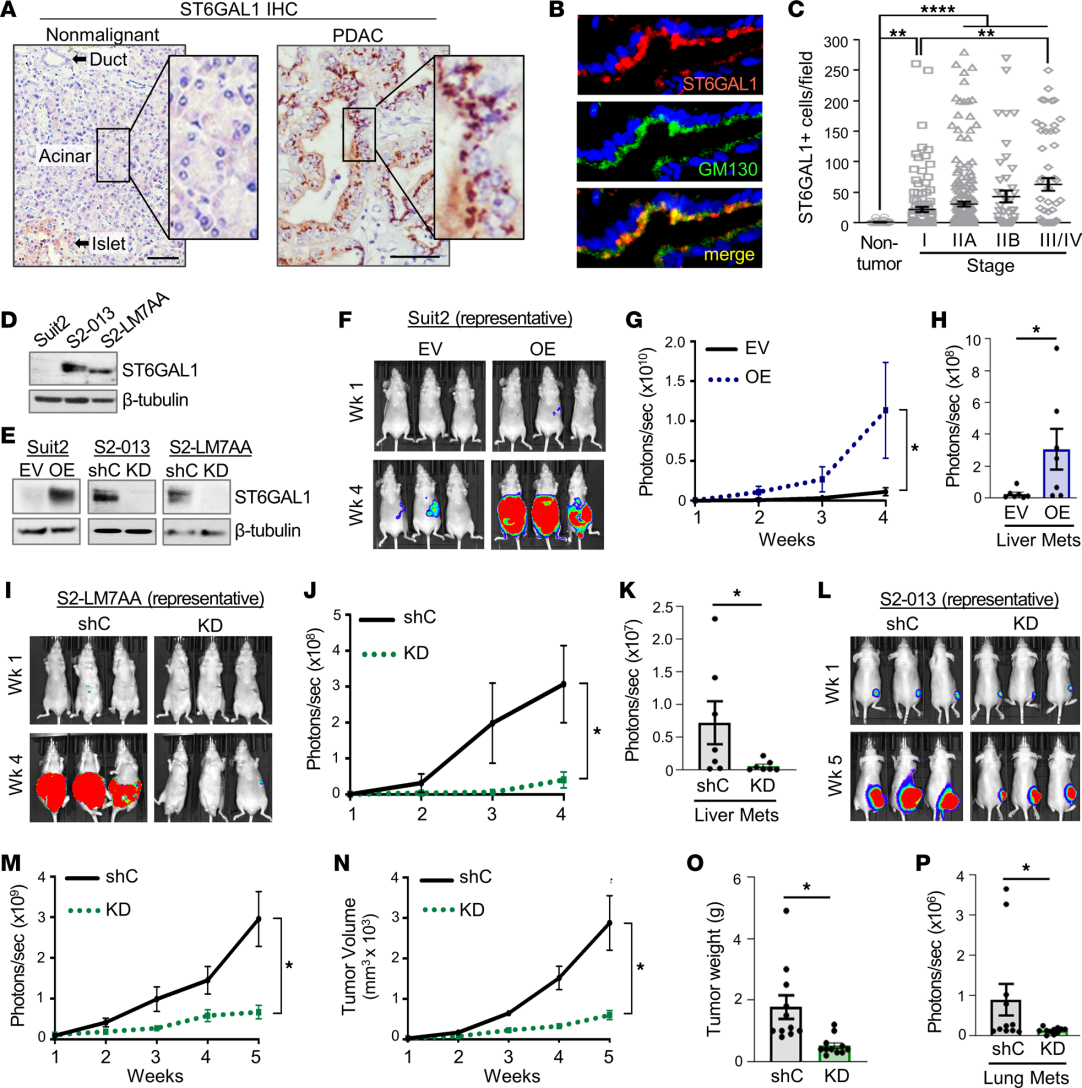
ST6GAL1 is upregulated in human PDAC and promotes tumor growth in xenograft models. (**A**) ST6GAL1 IHC in normal and malignant human pancreata. Scale bar: 100 μm; original magnification, 200×. (**B**) PDAC specimens costained for ST6GAL1 and the Golgi marker, GM130. (**C**) ST6GAL1-positive cells in human pancreatic tissues. Data analyzed by a Kruskal-Wallis test with follow-up analyses. For nontumor specimens, *n* = 48; stage I, *n* = 115; stage IIA, *n* = 216; stage IIB, *n* = 48; and stage III/IV, *n* = 55; **P < 0.001; ****P < 0.0001. (**D**) ST6GAL1 upregulation in the metastatic S2-013 and S2-LM7AA subclones relative to Suit2 parental cells. (**E**) ST6GAL1 was overexpressed (OE) in Suit2 cells or knocked down (KD) in the S2-013 and S2-LM7AA subclones. EV, empty vector control; shC, nontargeting shRNA control. (**F**) Representative bioluminescence imaging (BLI) of tumors formed from Suit2 EV and OE cells implanted into the pancreas. (**G**) Quantification of Suit2 EV and OE tumor growth by BLI (*n* = 7 mice/group). Results analyzed by 2-way ANOVA. *P < 0.05. (**H**) Livers were extracted from Suit2 EV and OE cohorts and imaged by BLI to detect metastases (*n* = 7). Data analyzed using a Mann-Whitney test, *P < 0.05. (**I**) Representative BLI of tumors formed from S2-LM7AA shC and KD cells implanted into the pancreas. (**J**) Quantification of S2-LM7AA shC and KD tumor growth by BLI (*n* = 7 mice/group). Two-way ANOVA, *P < 0.05. (**K**) Quantification of liver metastases by BLI (*n* = 7). Mann-Whitney test, *P < 0.05. (**L**) Representative BLI of tumors formed from S2-013 shC and KD cells implanted into the flank. (**M**) Quantification by BLI of flank tumors formed from S2-013 shC and KD cells (*n* = 11 mice/group). Two-way ANOVA, *P < 0.05. (**N**) Tumor volume was calculated from caliper measurements (*n* = 11). Two-way ANOVA, *P < 0.05. (**O**) Weights of tumors extracted at the endpoint (*n* = 11). Mann-Whitney test, *P < 0.05. (**P**) BLI quantification of metastatic tumors in the lungs (*n* = 11). Mann-Whitney test, *P < 0.05.

ST6GAL1 expression was evaluated by immunohistochemistry (IHC) in nonmalignant and PDAC patient pancreata (for antibody validation, see Supplemental Figure 1A; supplemental material available online with this article; https://doi.org/10.1172/jci.insight.161563DS1; and refs. 14, 25, 26, 39). In nonmalignant tissues, ST6GAL1 expression was undetectable in acinar cells and the great majority of ductal cells (Figure 1A), though a few ductal cells expressed ST6GAL1 (Supplemental Figure 1B). Interestingly, the islets stained positively for ST6GAL1 (Figure 1A and Supplemental Figure 1B), consistent with our prior identification of ST6GAL1 expression in β cells (14). In contrast with normal acinar cells, strong ST6GAL1 expression was observed in the malignant cells of patients with PDAC (Figure 1A and Supplemental Figure 1B). The expected Golgi localization of ST6GAL1 was verified by costaining for GM130 (Figure 1B). ST6GAL1 expression was elevated in early-stage PDAC (stage I), and levels were further increased in advanced stages (stages III/IV) (Figure 1C; representative IHC-stained tissues in Supplemental Figure 1C).

To interrogate a pro-tumorigenic function for ST6GAL1, tumor xenograft experiments were conducted using human Suit2 PDAC cells and 2 highly metastatic, isogenic Suit2 subclones generated through in vivo selection, S2-LM7AA and S2-013 (40, 41). Among PDAC cell lines, Suit2 cells have unusually low levels of ST6GAL1 (20). Intriguingly, the metastatic S2-LM7AA and S2-013 variants exhibited greatly upregulated ST6GAL1 (Figure 1D), suggesting selection for ST6GAL1 during metastatic progression. ST6GAL1 was overexpressed (OE) in Suit2 cells, or knocked down (KD) in S2-LM7AA and S2-013 cells (Figure 1E). Cells with ST6GAL1 OE had increased α2,6 sialylation, as indicated by staining with the SNA lectin, whereas α2,6 sialylation was decreased in cells with ST6GAL1 KD (Supplemental Figure 2A). Suit2 OE cells or empty vector (EV) controls were implanted into the pancreas, and tumor growth was monitored by bioluminescence imaging (BLI). Significantly increased tumor growth was observed for OE cells relative to EV cells (Figure 1, F and G), and OE cohorts had increased liver metastasis (Figure 1H, representative images in Supplemental Figure 2B). ST6GAL1 similarly promoted tumor growth in the S2-LM7AA cell model, evidenced by the reduced growth of pancreatic tumors (Figure 1, I and J) and decreased liver metastasis (Figure 1K) in the KD cohort compared with shC controls. For the S2-013 line, cells were injected subcutaneously into the flank and monitored for lung metastasis, as in other studies (41). Mice injected with S2-013 KD cells displayed reduced growth of flank tumors as indicated by BLI (Figure 1, L and M), tumor volume (Figure 1N), and tumor weight (Figure 1O). The KD cohort also had decreased lung metastasis (Figure 1P). Tumor growth and differential ST6GAL1 expression in tumors were verified by histology (Supplemental Figure 2, C and D). Additionally, tumors were evaluated for expression of phospho-EGFR (p-EGFR) and total EGFR (t-EGFR) (Supplemental Figure 2E). Together, xenograft experiments attest to ST6GAL1’s potent pro-tumorigenic activity.

### Ectopic expression of ST6GAL1 in mice expressing KRAS^G12D^ promotes accelerated PDAC progression and mortality.

**Figure 2:**
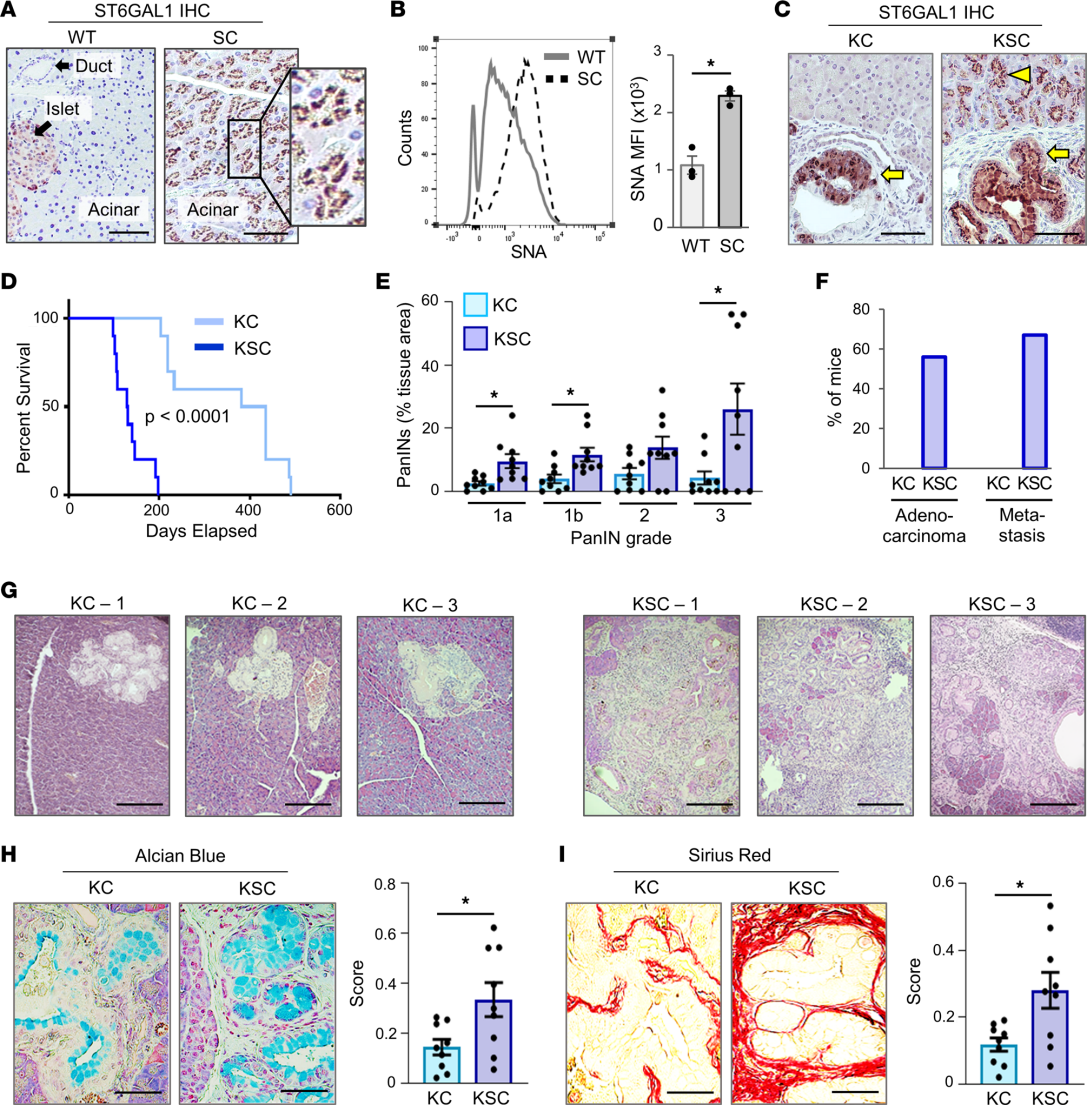
KSC mice exhibit accelerated PDAC progression and mortality compared with KC mice. (**A**) ST6GAL1 expression in the pancreas of SC mice (with *Pdx1-Cre*–driven expression of *ST6GAL1*), and WT mice (littermate controls expressing the *LSL-ST6GAL1* transgene, but not *Pdx1-Cre*). Scale bar: 100 μm; original magnification, 200×. (**B**) Cells dissociated from WT and SC pancreata were stained with SNA to detect surface α2,6 sialylation. Left: representative experiment; right: MFI values from 3 mice/genotype. Data analyzed using a Student’s *t* test. *P < 0.05. (**C**) ST6GAL1 IHC on KC and KSC pancreata depicting strong ST6GAL1 expression in PanINs (arrows). No detectable ST6GAL1 is noted in adjacent, normal KC acinar cells, whereas ST6GAL1 is highly expressed in normal KSC acinar cells (arrowhead), reflecting transgene expression. Scale bar: 100 μm. (**D**) Kaplan-Meier survival analysis indicates a median survival of 4.3 months for KSC mice and 13.6 months for KC mice (*n* = 10 mice/group). *P* < 0.0001. (**E**) H&E-stained pancreata from 20-week-old KC and KSC mice showing the percentage of overall tissue area represented by PanINs of varying grades (*n* = 9 mice/group). Data analyzed using a Student’s *t* test. *P < 0.05. (**F**) Percentage of 20-week-old KC and KSC mice that present with PDAC or distal metastases (*n* = 9 mice/group). (**G**) Representative pancreatic tissues showing more advanced disease in KSC mice (images from 3 individual mice/genotype). Scale bar: 200 μm. (**H**) Alcian blue staining for mucinous tumor cells in 20-week-old KC and KSC pancreata (*n* = 9 mice/group). Staining was quantified stereologically and analyzed using a Student’s *t* test. *P < 0.05. Scale bar: 100 μm. (**I**) Sirius red staining for collagen deposition in 20-week-old KC and KSC pancreata (*n* = 9 mice/group). Staining was quantified stereologically and data were analyzed using a Student’s *t* test. *P < 0.05. Scale bar: 100 μm. KC, *Pdx1-Cre LSL-Kras^G12D^*; KSC, *Pdx1-Cre LSL-ST6GAL1 LSL-Kras^G12D^*.

To model the upregulation of ST6GAL1 in PDAC, we generated mice with transgenic expression of human ST6GAL1. C57BL/6 mice were engineered with an *LSL-ST6GAL1* transgene inserted into the Rosa26 locus. These mice were crossed to the *Pdx1-Cre* line to direct pancreatic expression of ST6GAL1 (*Pdx1-Cre LSL-ST6GAL1*), hereafter abbreviated as SC mice. IHC confirmed ST6GAL1 expression in SC acinar cells (Figure 2A), whereas no detectable ST6GAL1 was observed in the acinar cells of littermate controls harboring the *ST6GAL1* transgene, but lacking Cre (*LSL-ST6GAL1*), referred to as WT for brevity. *Sambucus nigra* agglutinin (SNA) staining of cells dissociated from WT and SC pancreata revealed increased α2,6 sialylation of SC cells (Figure 2B). Necropsy performed on neonatal SC mice revealed no developmental abnormalities (Supplemental Figure 3). To study PDAC development, we used KC mice (42), in which *Pdx1-Cre* drives KRAS^G12D^ expression (*Pdx1-Cre LSL-Kras^G12D^*). The various lines were crossed to generate KSC mice, which express both KRAS^G12D^ and transgenic ST6GAL1 (*Pdx1-Cre LSL-ST6GAL1 LSL-Kras^G12D^*). No developmental abnormalities were observed in neonatal KC or KSC mice, although PanINs were detected at 8 weeks in both models (Supplemental Figure 4, A–F). Notably, endogenous ST6GAL1 was expressed in the PanIN lesions of KC mice (Figure 2C, arrow), illustrating an upregulation of ST6GAL1 in early stages of neoplasia. In KSC mice, ST6GAL1 was also expressed in PanINs; however, unlike KC mice, the adjacent, normal acinar cells displayed ST6GAL1 staining (Figure 2C, arrowhead), reflecting expression of the transgene.

A Kaplan-Meier survival analysis revealed dramatically accelerated mortality for KSC mice (Figure 2D), evidenced by a median survival of 4.3 months, as compared with 13.6 months for KC mice. The 13.6-month survival time for KC mice is comparable to that reported by others (43). Analyses of tissues harvested from the survival cohort verified pancreatic malignancy as the cause of mortality (Supplemental Figure 4G).

### ST6GAL1 promotes PanIN formation, adenocarcinoma, and metastasis.

To compare pathogenesis in age-matched mice, we examined tissues from 20-week-old KC and KSC mice. In the KC cohort, 2/9 mice had no apparent pancreatic abnormalities, whereas all 9 KSC mice displayed PanINs. KSC mice had more extensive overall PanIN development, and a particularly prominent increase was noted in PanIN3 (also referred to as high-grade dysplasia or carcinoma in situ) (Figure 2E). Additionally, 5/9 (56%) KSC mice developed adenocarcinoma (by histologic analysis), and 6/9 (67%) KSC mice had metastases to the liver, lungs, or other sites (Figure 2F, representative images in Supplemental Figure 4H). None of the KC mice developed adenocarcinoma or metastases at 20 weeks. H&E staining of pancreata indicated more advanced disease in KSC mice (Figure 2G), and staining with Alcian blue (Figure 2H) and Sirius red (Figure 2I) verified that KSC mice had a greater abundance of mucinous tumor cells and more extensive desmoplasia.

### RNA-Seq analyses of GEM pancreata indicate that ST6GAL1 activity upregulates stem- and cancer-associated gene networks, promotes a pancreatic ductal cell program, and induces activation of EGFR.

**Figure 3:**
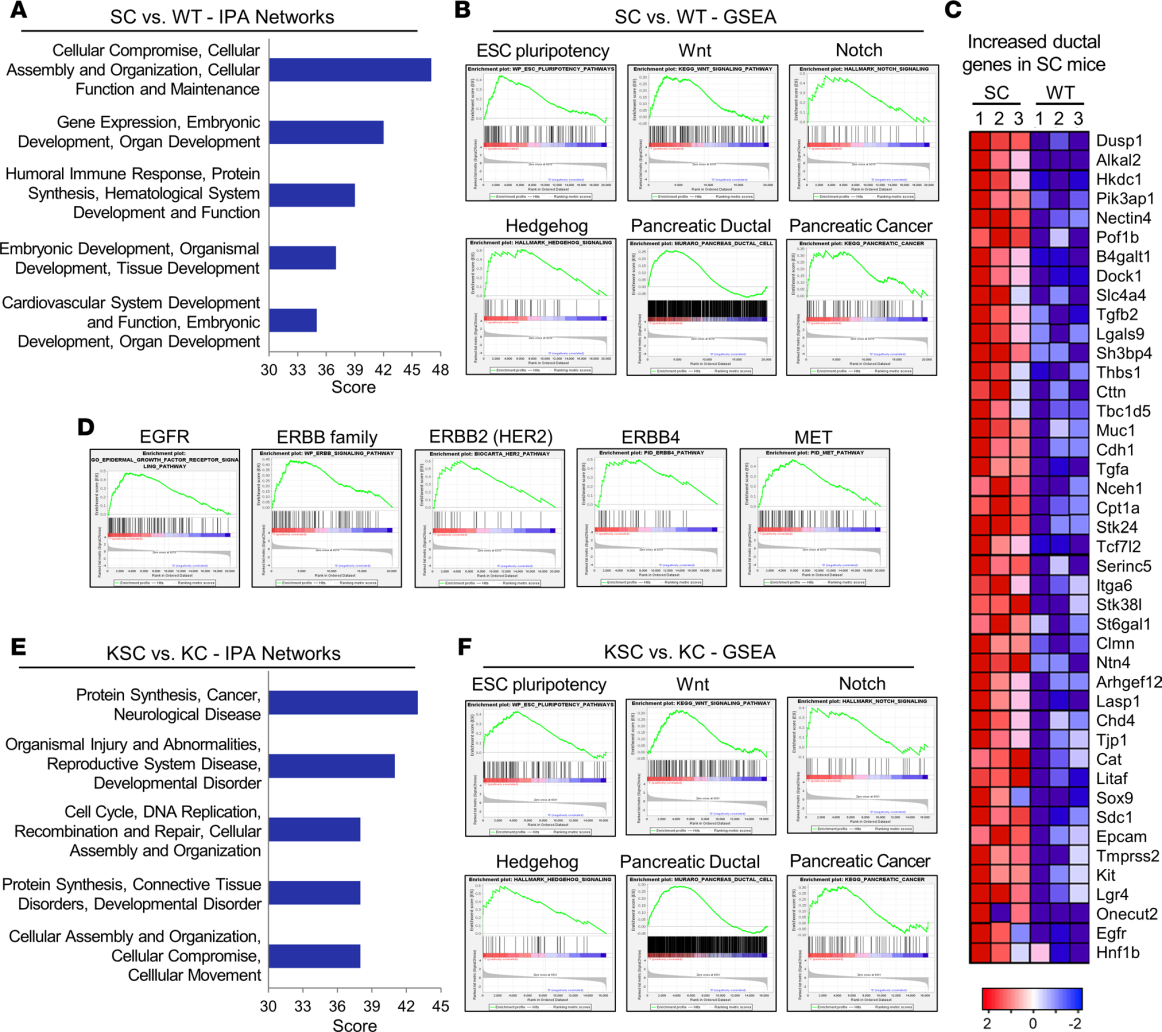
RNA-Seq of GEM pancreata reveals that ST6GAL1 promotes a stem and ductal phenotype and enhances activation of EGFR and other ERBB family members. (**A**) Ingenuity Pathway Analysis (IPA) of RNA-Seq data from 20-week-old SC and WT pancreata. The 5 top-scoring IPA networks altered in SC versus WT mice are shown (*n* = 3 mice/genotype). (**B**) GSEA indicates that compared with WT mice, SC mice have an upregulation in stemness networks (ESC pluripotency, Notch, Wnt, Hedgehog) as well as networks associated with a pancreatic ductal cell program and pancreatic cancer. NES and FDR values are as follows: ESC pluripotency: NES = 2.04, FDR = 0.011; Wnt: NES = 1.48, FDR = 0.103; Notch: NES = 1.70, FDR = 0.008; Hedgehog: NES = 1.89, FDR = 0.003; ductal: NES = 1.59, FDR = 0.018; pancreatic cancer: NES = 1.44, FDR = 0.135. NES, normalized enrichment score. (**C**) Heatmap of select genes from the pancreatic ductal network upregulated in SC mice (*n* = 3 mice/genotype). (**D**) GSEA reveals activation of EGFR and other receptor tyrosine kinases in SC mice. EGFR: NES = 2.22, FDR = 0.003; ERBB family: NES = 1.96, FDR = 0.015; ERBB2: NES = 1.96, FDR = 0.049; ERBB4: NES = 1.78, FDR = 0.023; MET: NES = 2.04, FDR = 0.010. (**E**) IPA of RNA-Seq data from 20-week-old KSC and KC pancreata depicting the 5 top-scoring IPA networks (*n* = 3 mice/genotype). (**F**) GSEA indicates that compared with KC mice, KSC mice have an upregulation in stemness-associated networks (ESC pluripotency, Notch, Wnt, and Hedgehog) and networks associated with a ductal phenotype and pancreatic cancer. NES and FDR values are as follows: ESC pluripotency: NES = 1.84, FDR = 0.033; Wnt: NES = 1.49, FDR = 0.123; Notch: NES = 1.38, FDR = 0.077; Hedgehog: NES = 2.09, FDR < 0.0005; ductal: NES = 1.65, FDR = 0.019; pancreatic cancer: NES = 1.26, FDR = 0.280.

Given ST6GAL1’s known role in promoting progenitor-like characteristics, we hypothesized that ectopic expression of ST6GAL1 in acinar cells may contribute to tumorigenesis by upregulating stem and ductal gene networks that facilitate both ADM and neoplastic transformation. To obtain an unbiased view of the effects of ST6GAL1, RNA-Seq was conducted on pancreata from 20-week-old WT and SC mice. At this age, there is no evidence of malignancy in SC mice. In the normal pancreas, acinar cells comprise 80%–90% of the organ mass; therefore, acinar transcripts dominate the mRNA pool (44). We used Ingenuity Pathway Analysis (IPA; QIAGEN) to identify the top 5 Associated Network Functions that differed between SC and WT mice (Figure 3A). Developmental processes were heavily represented, exemplified by embryonic development, organismal development, and organ development. Gene Set Enrichment Analyses (GSEAs) revealed that SC cells had an enrichment in developmental and stemness pathways, including ESC pluripotency, Wnt, Notch, and Hedgehog, and their associated downstream signaling molecules such as β-catenin (Wnt pathway) and the Hes/Hey axis (Notch pathway) (Figure 3B and Supplemental Table 1). The Wnt, Notch, and Hedgehog pathways play pivotal roles in pancreatic exocrine development, and PDAC is marked by reactivation of these pathways (45). Importantly, SC cells displayed elevated levels of canonical pancreatic ductal genes including *Sox9*, *Onecut2*, *Muc1*, *Hnf1b*, *Litaf*, and *Slc4a4* (Figure 3C). *Tgfa* and *Egfr* were also increased, which is noteworthy given the prominent role of TGF-α/EGFR signaling in ADM (37, 46, 47). GSEA verified increased activation of EGFR and other ERBB signaling networks in SC mice (Figure 3D), along with additional receptor tyrosine kinases such as MET (Figure 3D and Supplemental Table 1). MET and EGFR are activated in PanINs and PDAC lesions (4). Of particular interest, many cancer networks were elevated in SC mice, including pancreatic (Figure 3B), breast, renal, bladder, lung, and prostate (Supplemental Table 1). The activation of cancer-associated networks in SC cells aligns with the concept that ST6GAL1 upregulation induces molecular signaling events and transcriptomic changes that may predispose cells to neoplastic transformation.

We next compared 20-week-old KC and KSC mice. At this age, most KSC mice have advanced malignancy, whereas KC mice harbor mostly early-stage PanINs. Thus, many of the changes in gene expression are likely secondary to these divergent disease stages. IPA of KSC versus KC pancreata indicated that the top 5 Associated Network Functions related to cancer; developmental disorders; organismal injury and abnormalities; protein synthesis; and cellular compromise, assembly, and movement (Figure 3E). GSEA revealed that, compared with KC mice, KSC mice had an enrichment in stem cell networks (ESC pluripotency, Wnt, Notch, Hedgehog); EMT; a pancreatic ductal cell program; cancer-associated networks; and EGFR, ERBB2/HER2, and MET signaling pathways (Figure 3F and Supplemental Table 2).

### Nonmalignant acinar cells of mice with ectopic ST6GAL1 expression exhibit an upregulation in SOX9 and other ductal markers.

**Figure 4:**
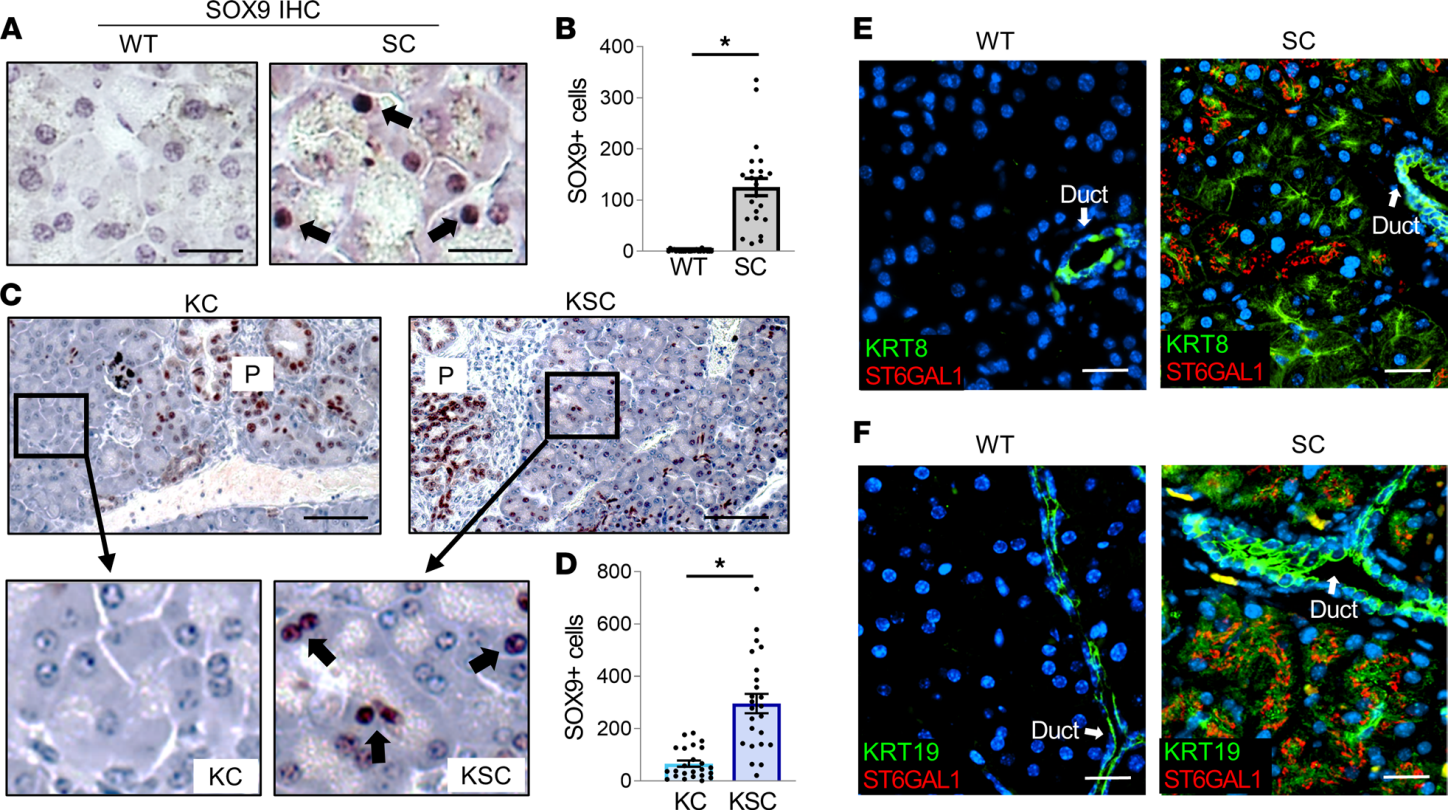
Acinar cells from mice with ectopic expression of ST6GAL1 have upregulated expression of SOX9 and other ductal markers. (**A**) IHC staining of pancreata from 20-week-old mice reveals SOX9 expression (arrows) in the acinar cells of SC, but not WT, mice. Scale bar: 20 μm. (**B**) SOX9-expressing acinar cells were quantified from IHC-stained SC and WT pancreata (*n* = 8 mice/genotype, with 3 tissue sections evaluated per mouse). Data analyzed using a Student’s *t* test. *P < 0.05. (**C**) IHC staining for SOX9 in pancreata from 20-week-old KC and KSC mice. “P” denotes PanINs, which are positive for SOX9. Scale bar: 100 μm; original magnification, 200×. Insets show upregulation of SOX9 (arrows) in the adjacent, normal-appearing acinar cells of KSC, but not KC, mice. (**D**) Quantification of SOX9-positive cells in the normal-appearing KC and KSC acinar cells (*n* = 8 mice/genotype, with 3 tissue sections evaluated per mouse). Data analyzed using a Student’s *t* test. *P < 0.05. (**E**) WT and SC pancreata were stained for ST6GAL1 (red) and cytokeratin 8 (KRT8, green) and counterstained with Hoechst (blue). KRT8 expression is observed in the acinar cells of SC, but not WT, mice. KRT8 is also expressed in the ductal cells of both WT and SC mice, as expected. Scale bar: 25 μm. (**F**) WT and SC pancreata were stained for ST6GAL1 (red) and cytokeratin 19 (KRT19, green) and counterstained with Hoechst (blue). KRT19 expression is observed in the acinar cells of SC, but not WT, mice. KRT19 is also expressed in the ductal cells of both WT and SC mice, as expected. Scale bar: 25 μm.

To verify activation of a ductal program in SC acinar cells, pancreata were IHC-stained for SOX9. SOX9 was not detected in WT acinar cells, in stark contrast with the large number of SOX9-positive SC acinar cells (Figure 4, A and B). As expected, SOX9 was expressed in normal ductal cells (Supplemental Figure 5). In KC and KSC mice, SOX9 was highly expressed in PanINs (“P”; Figure 4C), consistent with the known upregulation of SOX9 during malignant transformation (32). However, KSC, but not KC, mice displayed extensive SOX9 expression in adjacent, morphologically normal acinar cells (Figure 4, C and D). In addition to SOX9, SC acinar cells expressed other ductal markers including cytokeratins 8 and 19 (KRT8 and -19) (Figure 4, E and F). Thus, the ectopic expression of ST6GAL1 in acinar cells is sufficient to induce ductal gene expression. Interestingly, there were no obvious morphological differences between WT and SC acinar cells. Prior studies have suggested that during ADM-inducing events such as pancreatitis, mature acinar cells dedifferentiate through a series of stages (3). First, cells transition into an “acinar-like” cell with a normal morphology but increased expression of progenitor/ductal genes and decreased levels of some zymogens. This is followed by transition into a “progenitor-like” cell with more pronounced upregulation of ductal genes and downregulation of acinar genes. Finally, cells transdifferentiate into ductal-like cells with sharply reduced acinar markers and a tubular morphology characteristic of ADM lesions. We speculate that, in the absence of pancreatitis, ST6GAL1 activity confers a progenitor-like state in which acinar cells have enhanced stem and ductal gene expression but have not yet adopted a ductal-like morphology.

### Pancreatic organoids from mice with ectopic ST6GAL1 expression have enhanced growth and exhibit a gene expression profile consistent with a more progenitor-like state.

**Figure 5:**
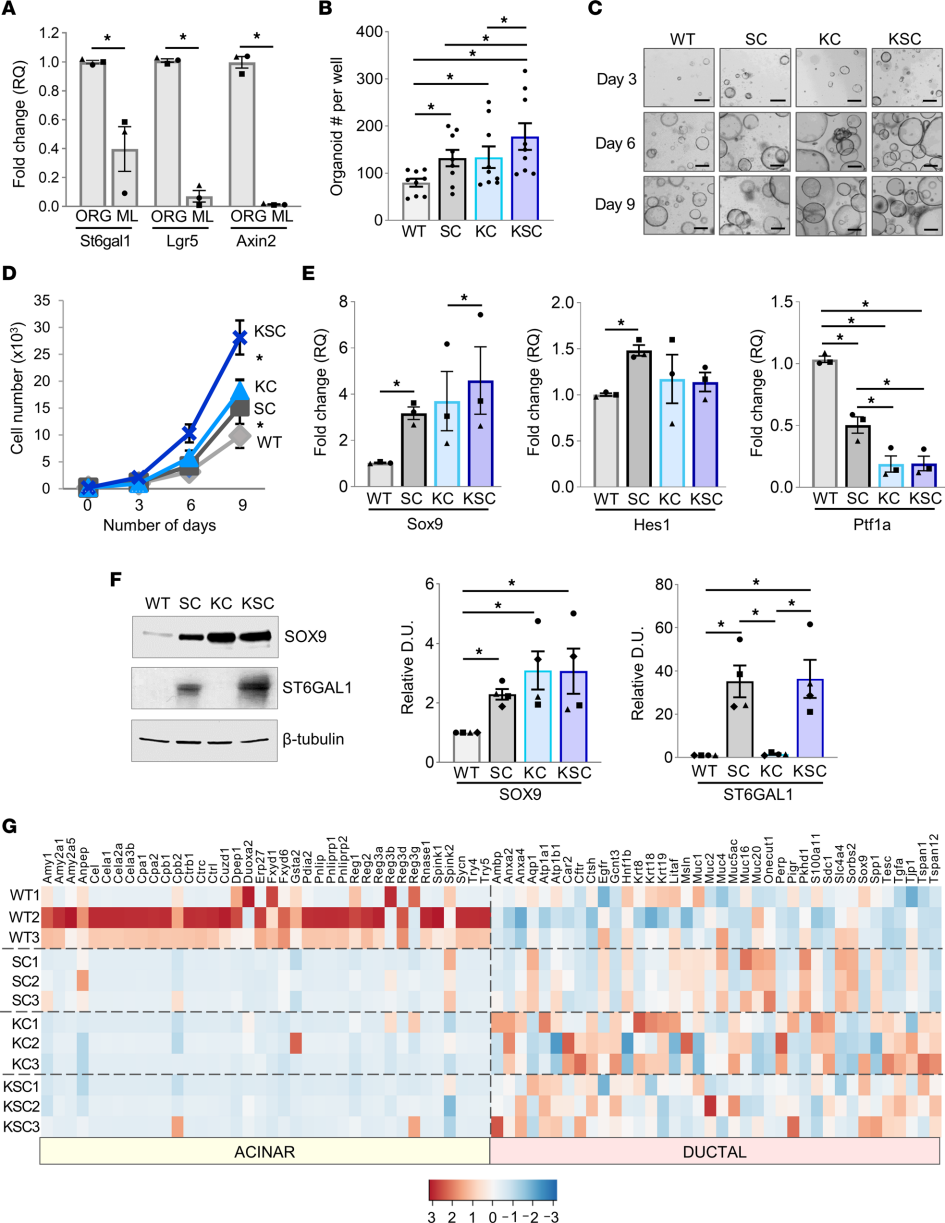
ST6GAL1 activity enhances the initiation and growth of GEM-derived organoids and promotes changes in gene expression consistent with a more progenitor-like state. (**A**) Cells dissociated from WT organoids were seeded into either organoid (ORG) culture, which maintains stemness properties, or monolayer (ML) culture in media with reduced stem cell factors, which induces cell differentiation. qRT-PCR analyses revealed that, similar to the stemness genes *Axin2* and *Lgr5*, endogenous *St6gal1* mRNA expression is downregulated in ML cultures (*n* = 3). Data analyzed using a Student’s *t* test. *P < 0.05. qRT-PCR, quantitative reverse transcriptase PCR; RQ, relative quantification. (**B**) Cells were dissociated from organoid lines and 2,000 cells seeded into fresh organoid culture. The number of organoids formed at day 3 was enumerated (*n* = 3 independent experiments, with 3 wells per genotype counted). Data analyzed by 1-way ANOVA, *P < 0.05. (**C**) Cells were dissociated from organoids, then seeded into fresh organoid culture, and organoid growth was monitored over time. Scale bar: 100 μm. (**D**) At days 3, 6, and 9 following the seeding of 2,000 organoid-derived cells into fresh organoid culture, organoids were dissociated, and the total number of cells was quantified (*n* = 4). Data analyzed using a Student’s *t* test. *P < 0.05. (**E**) *Sox9*, *Hes1*, and *Ptf1a* mRNA was quantified by qRT-PCR (*n* = 3). Data analyzed by 1-way ANOVA. *P < 0.05. (**F**) Organoids were lysed and immunoblotted for SOX9 and ST6GAL1. Densitometric analyses were conducted on blots from 4 independent organoid lysates. Data analyzed by 1-way ANOVA. *P < 0.05. Relative D.U., densitometric units normalized to β-tubulin. (**G**) RNA-Seq was conducted on organoid lines generated from 3 distinct mice per genotype. The heatmap depicts canonical acinar and ductal genes. Color key indicates *z* score.

Organoids were generated from the GEM models. Organoids initiate from progenitor-like cells and maintain progenitor properties during propagation in media containing stem cell factors (48). However, placing dissociated organoid cells into 2D confluent, monolayer culture in media with reduced stem cell factors causes cells to undergo differentiation (27, 49). Upon placement of cells from WT organoids into monolayer culture, endogenous *St6gal1* was downregulated, similar to the stemness genes, *Lgr5* and *Axin2* (Figure 5A).

We then compared organoids from the 4 GEM models for organoid-forming capability. The ability of dissociated, single cells to form organoids is an indicator of self-renewal potential. At 3 days following cell seeding into organoid culture, a greater number of organoids developed from SC compared with WT cells and KSC compared with KC cells (Figure 5B). Additionally, organoid growth over a 9-day interval was enhanced in the SC and KSC organoids relative to their WT and KC counterparts (Figure 5, C and D). The organoids were further examined for expression of the ADM-associated genes, *Sox9* and *Hes1*, and the differentiated acinar marker, *Ptf1a*. Compared with WT organoids, SC organoids had increased mRNA expression of *Sox9* and *Hes1* but reduced expression of *Ptf1a* (Figure 5E). Lineage tracing experiments have suggested that cells expressing SOX9, but not PTF1A, are capable of long-term clonal expansion from single cells (50). Comparing KC and KSC organoids, *Sox9* mRNA was increased in KSC organoids, but no significant differences were found in *Hes1* or *Ptf1a*, although *Ptf1a* expression was reduced in both the KC and KSC organoids relative to WT (Figure 5E). Immunoblots of organoid lysates verified that SC, KC, and KSC cells all expressed higher levels of SOX9 protein than WT cells (Figure 5F).

We next performed RNA-Seq on organoids. Three independent organoid lines per genotype were freshly generated, and RNA was harvested within the first 6–7 passages. As shown in Figure 5G, there was variability among the lines; however, one important finding was that WT organoids had conspicuously higher expression of mature acinar genes when compared with SC, KC, and KSC organoids. These data suggest that ST6GAL1 and KRAS^G12D^ can independently induce a shift toward a less-differentiated acinar cell phenotype. The expression of ductal genes was also variable; however, a subset of ductal genes appeared to be elevated in SC versus WT organoids, including *Aqp1*, *Onecut1*, *Pkhd1*, *Slc4a4*, *Spp1*, and several mucins. The KC and KSC organoids had more pronounced increases in ductal genes relative to WT organoids. The reason why alterations in acinar gene expression were more dramatic than noted for ductal genes is not currently understood. However, it should be mentioned that cells in organoid culture are, in fact, more ductal and progenitor-like than acinar cells found within the intact pancreas, given that organoid culture selects for progenitor cells, and organoids are grown in the presence of stem cell factors. Both acinar and ductal cells can initiate organoid formation; however, only the ductal-like cells are capable of growth in organoid culture over extended passages (51).

### Knockdown of ST6GAL1 in KC organoids suppresses organoid growth and ductal gene expression while increasing acinar gene expression.

**Figure 6:**
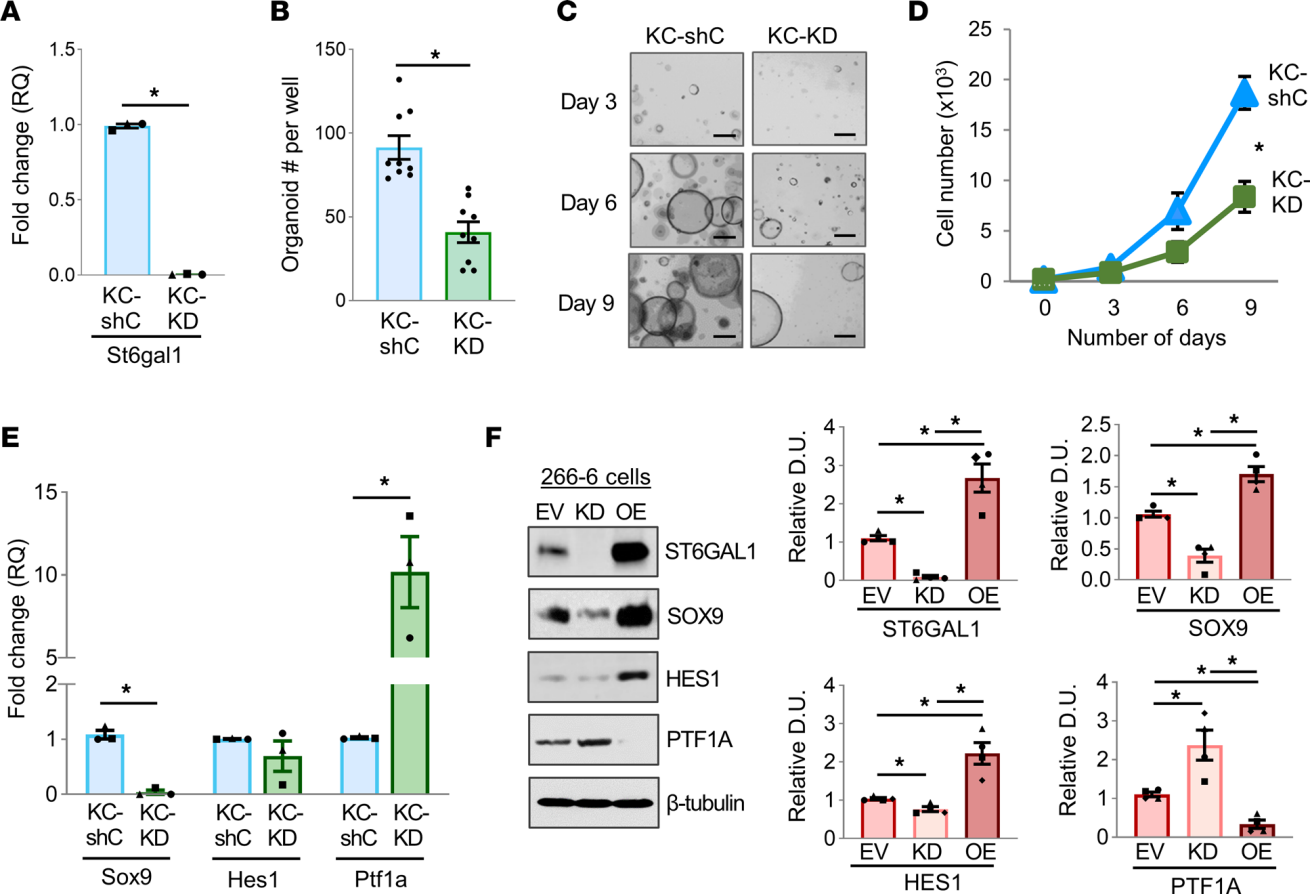
ST6GAL1 activity is important for a progenitor-like phenotype in KC organoids and in the 266-6 cell ADM model. (**A**) KC organoids were transduced with lentivirus encoding *St6gal1* shRNA (KC-KD) or a control shRNA vector (KC-shC). Knockdown of *St6gal1* was verified by qRT-PCR (*n* = 3). Data analyzed using a Student’s *t* test. *P < 0.05. (**B**) Cells were dissociated from the KC-shC and KC-KD organoids, and 2,000 cells were seeded into fresh organoid culture. The number of organoids formed at day 3 was enumerated (*n* = 3 independent experiments with 3 wells counted per genotype). Data analyzed using a Student’s *t* test. *P < 0.05. (**C**) Images of the KC-shC and KC-KD organoids. Scale bar: 100 μm. (**D**) At days 3, 6, and 9, organoid cultures were dissociated, and the total number of cells was quantified (*n* = 4). Data analyzed using a Student’s *t* test. *P < 0.05. (**E**) *Sox9*, *Hes1*, and *Ptf1a* mRNA was quantified by qRT-PCR (*n* = 3). Data analyzed using a Student’s *t* test. *P < 0.05. (**F**) ST6GAL1 was overexpressed (OE) or knocked down (KD) in the 266-6 pancreatic cancer cell line (EV, empty vector control). The expression of SOX9, HES1, and PTF1A was measured by immunoblotting. Graphs depict densitometric analyses of blots from 4 independent lysates. Data analyzed using a Student’s *t* test. *P < 0.05.

To determine whether ST6GAL1 was important for maintaining progenitor characteristics in cells expressing KRAS^G12D^, ST6GAL1 expression was knocked down in KC organoids (KC-KD, Figure 6A). ST6GAL1 KD inhibited organoid formation (Figure 6B) and organoid growth over time (Figure 6, C and D). Moreover, KC-KD organoids had decreased expression of *Sox9* and markedly increased *Ptf1a* (Figure 6E) compared with KC-shC controls, suggesting that ST6GAL1 KD reverted KRAS^G12D^-expressing cells to a more differentiated acinar phenotype. Thus, for both nonmalignant (WT/SC) and neoplastic (KC) organoids, ST6GAL1 activity was important for organoid growth and the maintenance of a ductal gene program.

### ST6GAL1 promotes the expression of ductal markers in the 266-6 ADM cell model.

The acinar-like 266-6 pancreatic cancer cell line is a well-established model for studying ADM (52). Accordingly, 266-6 cells with ST6GAL1 OE or KD were evaluated for SOX9, HES1, and PTF1A expression by immunoblotting. Relative to EV controls, ST6GAL1-KD cells had reduced SOX9 and HES1, and increased PTF1A, whereas ST6GAL1-OE cells had increased SOX9 and HES1 expression but sharply reduced PTF1A (Figure 6F).

### ST6GAL1 activity enhances pancreatitis-induced ADM.

**Figure 7:**
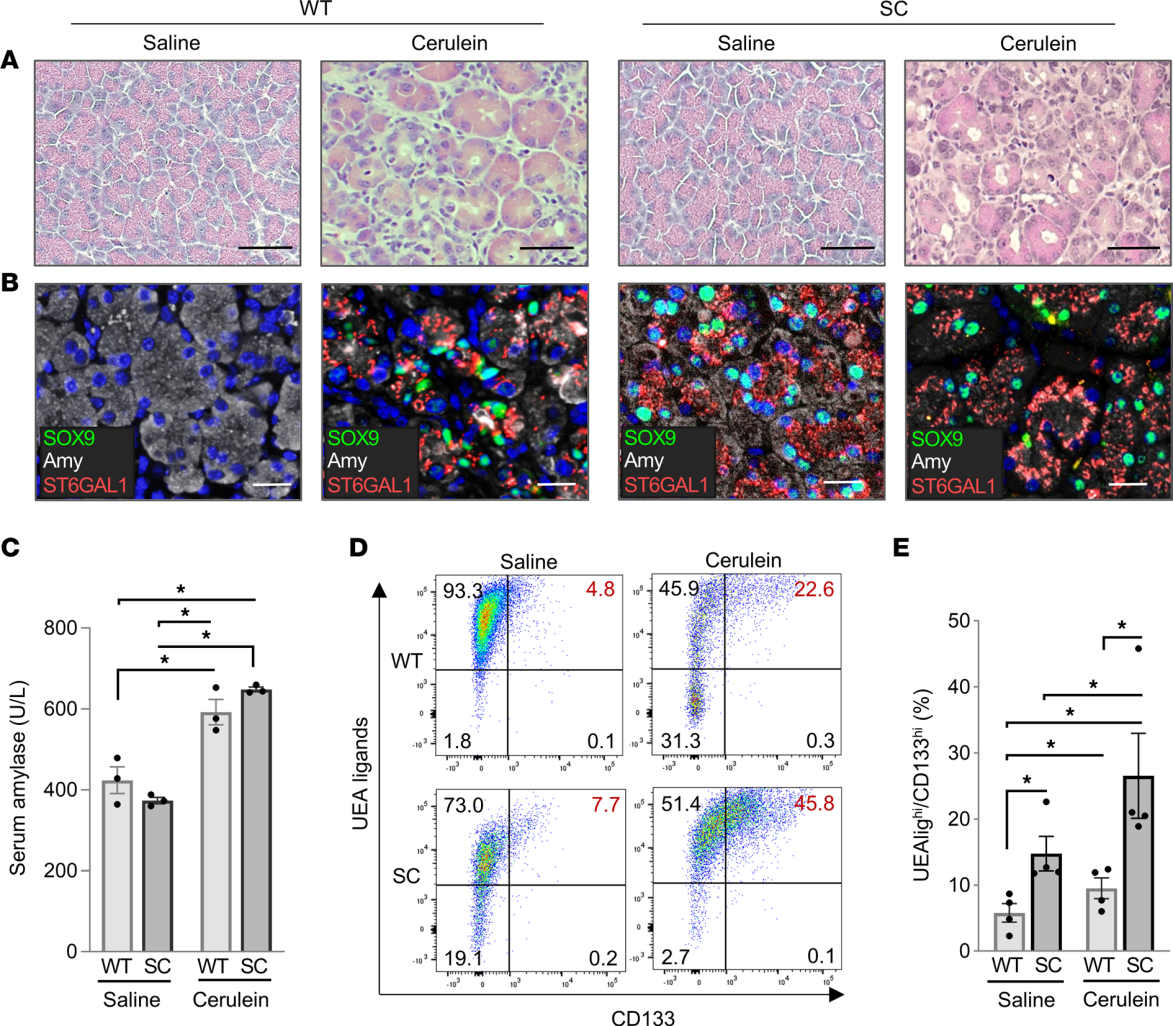
ST6GAL1 promotes ADM in the cerulein-induced pancreatitis model. (**A**) WT and SC mice were injected i.p. with saline (control) or cerulein to induce pancreatitis. Cerulein-induced damage to the pancreas was confirmed by H&E. Scale bar: 50 μm. (**B**) Pancreata from mice injected with saline or cerulein were evaluated for coexpression of SOX9 (green), ST6GAL1 (red), and the acinar marker, amylase (white; note that amylase is expressed throughout the cytosol, consistent with localization to zymogen granules). Nuclei were stained with Hoechst (blue). In WT mice (left panels), ADM-like cells coexpressing ST6GAL1, SOX9, and amylase were identified in the cerulein-treated, but not saline-treated, cohorts. In SC pancreata (right panels), SOX9-positive acinar cells were detected in both the saline- and cerulein-treated mice. Scale bar: 25 μm. (**C**) Mice injected with cerulein had increased serum amylase levels, verifying induction of pancreatitis (*n* = 3 mice/group). Data analyzed by 2-way ANOVA. *P < 0.05. (**D**) Cells were dissociated from the pancreata of WT and SC mice treated with saline or cerulein. Flow cytometry was conducted on cells stained with UEA lectin (acinar marker), anti-CD133 (ductal marker), anti-EpCAM (epithelial marker), anti-CD45 (immune marker), and Aqua live/dead stain (marker for nonviable cells). UEA and anti-CD133 staining was quantified on viable, singlet epithelial cells (EpCAM positive, CD45 negative, Aqua dye negative). Cells undergoing ADM coexpress UEA ligands and CD133. (**E**) Quantification of cells undergoing ADM (UEAlig^hi^/CD133^hi^) in cerulein- or saline-treated mice (*n* = 4 mice/group). Data analyzed by 2-way ANOVA. *P < 0.05.

Pancreatitis is a potent inducer of ADM; we therefore examined ST6GAL1 activity in the cerulein-stimulated pancreatitis model (52). Upon injection of WT and SC mice with cerulein, acinar cells adopted a tubular-like morphology characteristic of inflammatory damage and ductal transdifferentiation (Figure 7A). Tissues were stained for amylase (acinar marker), SOX9, and ST6GAL1 (Figure 7B). No detectable SOX9 or ST6GAL1 was observed in healthy WT acinar cells (saline control), whereas numerous cells within WT pancreatitis tissues (cerulein) coexpressed amylase, SOX9, and ST6GAL1. These data show that pancreatitis induces an upregulation in endogenous ST6GAL1 and that ST6GAL1 expression is found specifically in the ADM-like cells. In SC mice, SOX9 was highly expressed in the acinar cells of both the healthy and inflamed pancreas, indicating that ST6GAL1 activity is sufficient to induce SOX9 expression. The induction of pancreatitis by cerulein was verified by measuring serum amylase levels (Figure 7C).

The number of ADM-like cells was quantified in WT and SC mice by staining dissociated pancreatic cells for surface markers of ADM. Acinar cells express fucosylated glycans recognized by the *Urex europaeus* agglutinin (UEA) lectin; however, they have low expression of CD133. Conversely, ductal cells express high CD133 but low levels of UEA ligands. Cells undergoing ADM have high expression of both UEA ligands and CD133 (UEA-lig^hi^CD133^hi^) (53). For the cerulein-treated cohorts, significantly more UEA-lig^hi^CD133^hi^ cells were found in SC versus WT pancreata, indicating that ST6GAL1 expression in acinar cells promotes ADM within an inflammatory milieu (Figure 7, D and E). Moreover, more UEA-lig^hi^CD133^hi^ cells were present within the healthy (saline) SC pancreas compared with healthy WT pancreas (Figure 7E), in congruence with our other data suggesting ST6GAL1 promotes progenitor characteristics even in the absence of inflammation.

### EGFR is activated in acinar cells with ectopic ST6GAL1 expression.

**Figure 8:**
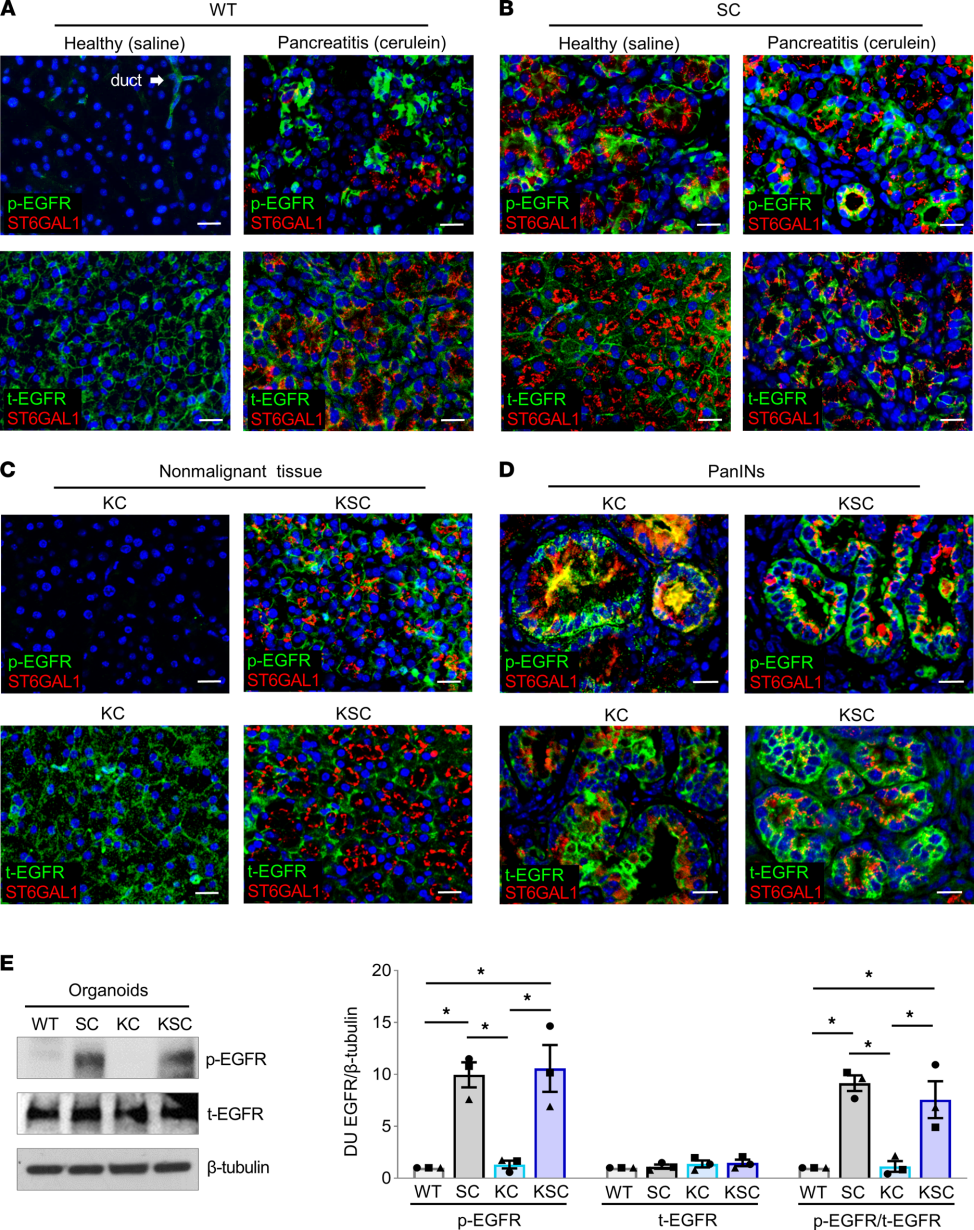
EGFR is activated in pancreatic tissues and organoids from mice with ectopic expression of ST6GAL1. (**A**) Upper panels: Healthy (saline) and pancreatitis (cerulein) WT tissues were stained for ST6GAL1 (red) and p-EGFR (pY-1068, green). Lower panels: Tissues stained for ST6GAL1 (red) and t-EGFR (green). Nuclei were stained with Hoechst. The acinar cells of healthy WT tissues lack detectable p-EGFR, whereas weak p-EGFR staining is noted in WT ductal cells (arrow). EGFR is strongly activated in WT acinar cells in pancreatitis tissues. Furthermore, endogenous ST6GAL1 is upregulated in WT acinar cells exposed to pancreatitis. Scale bar: 25 μm. (**B**) Upper panels: SC healthy and pancreatitis tissues were stained for ST6GAL1 (red) and p-EGFR (pY-1068, green). Lower panels: Tissues stained for ST6GAL1 (red) and t-EGFR (green). Nuclei were stained with Hoechst. Robust expression of p-EGFR is observed in SC acinar cells in both healthy and pancreatitis tissues. Scale bar: 25 μm. (**C**) Upper panels: Adjacent, nonmalignant tissues from KC and KSC mice were stained for ST6GAL1 (red) and p-EGFR (green). Lower panels: Nonmalignant tissues were stained for ST6GAL1 (red) and t-EGFR (green). EGFR was activated in the nonmalignant acinar cells of KSC, but not KC, mice. Scale bar: 25 μm. (**D**) Upper panels: PanIN lesions were stained for ST6GAL1 (red) and p-EGFR (green). Lower panels: PanINs were stained for ST6GAL1 (red) and t-EGFR (green). EGFR was activated in the PanINs of both KC and KSC mice. Additionally, endogenous ST6GAL1 was upregulated in the PanINs of KC mice. Scale bar: 25 μm. (**E**) Organoid lysates were immunoblotted for p-EGFR (pY-1068) and t-EGFR. Graphs depict densitometric units (D.U.) measured on blots from 3 independent organoid lysates. D.U. were normalized to values for β-tubulin. Data analyzed by 1-way ANOVA. *P < 0.05.

To investigate the role of receptor sialylation in acinar reprogramming, we focused on EGFR given its known activation by ST6GAL1 and pivotal role in ADM. In healthy WT pancreata, acinar cells were devoid of p-EGFR, although a low level of p-EGFR was apparent in the ducts (Figure 8A). However, in the inflamed pancreas, WT acinar cells displayed strong activation of EGFR, consistent with the literature (54), and this was accompanied by an upregulation in ST6GAL1 (Figure 8A). Strikingly, the acinar cells in healthy SC pancreata displayed robust activation of EGFR, at levels comparable to those found in SC pancreatitis tissues (Figure 8B). We then evaluated EGFR activation in KC and KSC pancreata. In nontumor regions, the normal-appearing acinar cells of KSC, but not KC, mice had high levels of activated EGFR (Figure 8C), similar to SC acinar cells. On the other hand, EGFR was strongly activated in the PanINs of both KC and KSC mice (Figure 8D), in line with other reports (55). Finally, we examined EGFR activation in organoids. Pronounced activation of EGFR was detected in SC and KSC organoids but not in WT and KC organoids (Figure 8E). Collectively, these results suggest that the sialylation-dependent activation of EGFR may constitute an important mechanism by which ST6GAL1 activity reprograms acinar cells into a more ductal, progenitor-like state.

## Discussion

Cancer-associated changes in glycosylation have been reported for decades, and certain glycan structures, such as sialyl Lewis a (sLe^a^, CA19-9), are used in the clinic to monitor cancer progression and recurrence (56). Nonetheless, glycans and glycosyltransferases remain largely unexplored as potential biomarkers or therapeutic targets. Accumulating evidence points to an important functional role for aberrant sialylation in pancreatic and other cancers (57). Mice engineered to express sLe^a^ in combination with KRAS^G12D^ developed more aggressive pancreatic cancer compared with mice expressing KRAS^G12D^ alone (58). Sialylation by ST6GAL1 has likewise been previously implicated in pancreatic cancer. An increase in α2,6 sialylation was one of the major glycan changes detected in the neoplastic tissues of PDAC patients and KC mice, and deletion of *St6gal1* in KC mice impeded PanIN formation (59). Additionally, Hsieh et al. observed that fructose administration to cerulein-treated KC mice accelerated invasive PDAC, and ST6GAL1 was central to this process (60). The present investigation reinforces prior work by establishing a tumor-promoting function for ST6GAL1 in tumor xenograft and GEM models. Indeed, the combined expression of transgenic ST6GAL1 and KRAS^G12D^ in KSC mice reduced median survival to 4.3 months, an interval comparable to the survival time of the aggressive KPC PDAC model, which expresses KRAS^G12D^ and mutant p53 (*Trp53^R172H^*) (43).

ST6GAL1 endows cancer cells with stemness features including tumor-initiating potential in limiting dilution assays (25); we therefore postulated ST6GAL1 may facilitate early stages in PDAC development. We find that ST6GAL1 is upregulated in PanINs and stage I PDAC, with levels increasing in advanced-stage malignancy. Alexander et al. similarly observed a progressive upregulation of ST6GAL1 during the transition between gastric premalignancy and gastric cancer, and in this same report, ST6GAL1 was identified as a marker for gastric stem cells (27). ST6GAL1 contributes functionally to stemness-associated processes, including EMT, iPSC reprogramming, and acquisition of CSC characteristics (20, 25, 26, 29, 61). Our RNA-Seq results comparing SC and WT pancreata highlighted a role for ST6GAL1 in activating stemness/developmental pathways, including Wnt, Notch, and Hedgehog. These same pathways were activated when ST6GAL1 was overexpressed in Suit2 PDAC cells (20). The Wnt, Notch, and Hedgehog cascades are well-known players in PDAC development (45, 62, 63), and Notch cooperates with oncogenic KRAS in directing ADM and PanIN formation (64). Also, signaling by Wnt and Notch during ADM suppresses the expression of acinar-specific genes (31). The Notch pathway is activated in acinar cells downstream of EGFR signaling. EGFR activation in ADM-like cells induces an upregulation in protein kinase D1, which in turn, activates the Notch signaling node (31, 65).

The source of stem/progenitor cells in the adult exocrine pancreas remains controversial, and a bona fide stem cell population has yet to be defined. Alternatively, acinar cells are highly plastic, and some acinar subpopulations function as facultative progenitors when tissue regeneration is needed (66, 67). Acinar cells undergoing ADM are a major source of facultative progenitors, and these cells also serve as tumor-initiating cells (66, 67). While studies have indicated that PDAC can initiate from either acinar or ductal cells, recent evidence suggests that acinar cells are much more susceptible to transformation by KRAS (32, 68). The dedifferentiation of acinar cells during ADM further sensitizes cells to KRAS-driven oncogenesis (69, 70). Data herein show that endogenous ST6GAL1 is upregulated in the ADM-like cells induced by pancreatitis. Moreover, SC mice form quantitatively more ADM-like cells during pancreatitis than WT mice, suggesting a causal role for ST6GAL1 in promoting inflammation-associated ADM. Importantly, the ectopic expression of ST6GAL1 in acinar cells induces a pronounced upregulation in a ductal gene network (in the absence of either pancreatitis or the KRAS oncogene), as evidenced by RNA-Seq results and staining of SC tissues for SOX9, KRT8, and KRT19. These data align with our prior studies indicating that ST6GAL1 activity enhances SOX9 expression in many cancer cell models (25). Additionally, ST6GAL1 promotes the expression of SOX9, while suppressing PTF1A, in both the classic 266-6 ADM cell line and GEM-derived organoids. Also important, the initiation and growth of both nonmalignant and KRAS^G12D^-expressing organoids is dependent upon ST6GAL1 activity. Clonogenic growth of primary cells in organoid models is a key feature of stem/progenitor cell behavior (71).

ST6GAL1 regulates intracellular signaling, and consequently, gene expression, by sialylating receptors such as EGFR. The activation of EGFR by TGF-α is one of the primary mechanisms responsible for driving ADM (35, 36) and EGFR signaling induces SOX9 expression (34). EGFR promotes acinar transdifferentiation into ductal-like cells both in vitro (47) and in vivo (37, 38). Additionally, EGFR signaling is essential for KRAS-driven PDAC development (55, 72). EGFR activates WT KRAS and thereby collaborates with mutant *KRAS* alleles to amplify KRAS-dependent pathways (2, 73). We previously reported that ST6GAL1 sialylates and activates EGFR in PDAC, ovarian, and colon cancer cell lines, as well as in nonmalignant cells (19–22), although an inhibitory effect of sialylation has also been reported (74, 75). Mechanistically, ST6GAL1-mediated sialylation of EGFR promotes (i) formation of the active EGFR dimer, (ii) EGFR-dependent AKT and NF-κB activation, (iii) recycling of EGFR to the cell surface, and (iv) protection against lysosome-mediated EGFR degradation (22). It is well established that N-glycans play seminal roles in modulating EGFR structure and signaling (76–78). For example, an N-glycan attached to Asn579 is crucial for maintaining the autoinhibitory tether within EGFR (79), and this N-glycan is a target for sialylation by ST6GAL1 (80). Deletion of the Asn579 N-glycan releases the tether, leading to EGFR activation (79). One speculative hypothesis is that the addition of the bulky, negatively charged sialic acid to the terminus of this N-glycan disrupts the tether, promoting EGFR activation. While future research will be needed to delineate the specific effects of α2,6 sialylation on the overall structure of EGFR, it is noteworthy that SC acinar cells and SC-derived organoids display a dramatic enrichment in EGFR activation when compared with WT acinar cells and organoids. These data point to a potential mechanism by which ST6GAL1-mediated receptor sialylation may promote the upregulation of stem and ductal gene networks.

In summary, results from this investigation suggest that ST6GAL1 activity reprograms mature acinar cells to adopt a more ductal, progenitor-like phenotype that poises cells for ADM. When occurring in conjunction with an oncogenic event, such as a *KRAS* mutation, this may facilitate neoplastic transformation, as well as the transition to malignancy. Extensive literature has documented ST6GAL1 overexpression in a plethora of human cancers; however, knowledge regarding ST6GAL1’s functional contribution to carcinogenesis remains limited. The collective findings in this report provide important insights into the mechanisms by which ST6GAL1 imparts cell characteristics that foster neoplastic development.

## Methods

### ST6GAL1 IHC on human pancreatic tissues

Tissue microarrays containing nonmalignant and PDAC patient tissues were obtained from US Biomax Inc. (PA1001c, PA2072a, PA1921, PA1002b). Sections were IHC-stained for ST6GAL1 as reported (25, 26). Briefly, sections were subjected to antigen retrieval using Antigen Unmasking Solution, Citric Acid Based (Vector Laboratories); blocked with 2.5% horse serum for 1 hour; and incubated overnight at 4°C with goat polyclonal antibody against ST6GAL1 (see Supplemental Table 3 for antibody information). Sections were incubated with ImmPRESS-HRP anti-goat IgG for 1 hour and developed using ImmPACTNovaRED or ImmPACTDAB (Vector Laboratories). Images were captured with a Nikon 80i Eclipse microscope and processed with Nikon NIS-Elements imaging software.

### Cell culture

Suit2 and S2-013 cell lines were a gift from Michael Hollingsworth (University of Nebraska, Omaha, Nebraska, USA). The S2-LM7AA line was developed by Lacey McNally and Donald Buchsbaum (University of Alabama at Birmingham, UAB). Suit2 EV and OE cells, and S2-013 and S2-LM7AA shC and KD cells, were generated using lentivirus and propagated in RPMI media (Thermo Fisher Scientific) as previously reported (20). Murine 266-6 cells (ATCC CRL-2151) were transduced with lentivirus encoding *St6gal1* (Genecopoeia, LPP-EX-Mm05221-Lv105) or shRNA against *St6gal1* (MilliporeSigma, TRCN00000018818). Murine 266-6 cells were grown on gelatin-coated flasks (Corning) and cultured in DMEM containing 10% FBS and an antibiotic/antimycotic supplement (Thermo Fisher Scientific). All cell lines represent stable polyclonal populations.

### Tumor xenograft experiments using the Suit2 isogenic series

Luciferase was stably expressed in Suit2, S2-013, and S2-LM7AA cells using lentivirus (Cellomics Technologies). For Suit2 and S2-LM7AA cells, a suspension of 1 × 10^6^ cells in 20 μL of PBS was injected into the pancreas of athymic nude mice (Jackson Laboratory). Tumor growth was monitored by BLI using the IVIS Lumina Series III (PerkinElmer). At the endpoint, extracted livers were imaged by BLI. For the S2-013 line, 1 × 10^6^ cells suspended in 500 μL PBS containing 10% Matrigel were injected into the flank. Tumor growth was monitored by BLI and tumor volume calculated from caliper measurements (L × W × H). At the endpoint, excised tumors were weighed and lungs imaged by BLI. For Suit2 and S2-LM7AA experiments, *n* = 7 mice/group, and for S2-013, *n* = 11 mice/group.

### GEM models

To develop SC mice, an *LSL-ST6GAL1* transgene under control of the *Rosa26* promoter was inserted into the *Rosa26* locus of C57BL/6 mice as described (25). Mice expressing the *LSL-ST6GAL1* transgene were crossed to *Pdx1-Cre* mice. KC mice were obtained by crossing mice expressing *LSL-Kras^G12D^* with the *Pdx1-Cre* line (the *LSL-Kras^G12D^* and *Pdx1-Cre* lines were obtained from the Jackson Laboratory). KSC mice were obtained by crossing SC mice (*Rosa26-LSL-ST6GAL1 Pdx1-Cre*) with *LSL-Kras^G12D^* mice. Genotype was verified by PCR using the following primers: Kras mutant Forward: 5′ AAGCTAGCCACCATGGCT 3′; Kras Reverse: 5′ CGCAGACTGTAGAGCAGCG 3′; Cre Forward: 5′ TGCCACGACCAAGTGACAGC 3′; Cre Reverse: 5′ CCAGGTTACGGATATAGTTCATG 3′; ST6GAL1 Forward: 5′ CCAGGACCAGGCATCAAGTT 3′; ST6GAL1 Reverse: 5′ CCCATAGCTCCCAAGGCATC 3′.

Necropsy performed on neonatal mice by a veterinary pathologist revealed no developmental abnormalities (Supplemental Figures 3 and 4). Additionally, SC mice had normal weights and blood glucose levels. ST6GAL1 transgene expression was confirmed by IHC, and increased α2,6 sialylation was validated by staining SC cells with SNA. For SNA staining, pancreata were digested using Miltenyi Tissue Dissociation Kit (Miltenyi Biotec), and dispersed cells were passed through a 100 μm filter (Thermo Fisher Scientific). Cells were resuspended in PBS containing 10 μM ROCK inhibitor (Hello Bio) to improve viability. Cells were stained for 30 minutes on ice with Aqua live/dead stain, anti-EpCAM–PE, and SNA-FITC (reagent information in Supplemental Table 3). MFI values for SNA staining were measured on viable cells (Aqua negative) that were positive for EpCAM (epithelial marker). Unstained and single-color controls were used for compensation and gating, and only viable singlets were included. A BD LSR II flow cytometer (BD Biosciences) was used to acquire data, and FlowJo V10 was used for data analysis. Experiments were conducted on 3 mice per group.

For survival analyses, mice were euthanized when they lost 20% of body weight and/or presented with low body score (*n* = 10 mice/genotype). Tissues were evaluated for macro-metastases in lungs, liver, intestines, peritoneum, and other organs, and micro-metastases were identified by examining H&E-stained sections.

### Organoids

As described before (49), pancreata were minced and placed in collagenase I solution (Gibco) for 45 minutes with intermittent trituration. Samples were passed through a 70 μm cell strainer (Thermo Fisher Scientific), and after washing, dispersed cells were resuspended in Matrigel (Corning). A total of 15 μL of the cell suspension was placed in a 24-well plate. The plate was inverted and the Matrigel allowed to solidify at 37°C for 12 minutes. Subsequently, cultures were grown for 3 days in organoid media (81), specifically, DMEM containing 50% L-WRN–conditioned media with the following supplements: 10 μM ROCK inhibitor (Hello Bio), 10 μM TGF-βR1 inhibitor (Selleck Chemicals), and 10 mM nicotinamide (LKT Labs). Organoids were passaged until free of contaminating cells such as fibroblasts, then used for experiments.

For some experiments, organoid-derived cells were placed into adherent monolayer culture. Tissue culture wells were coated with Matrigel (1:30 dilution in PBS), and the Matrigel was allowed to solidify for 30 minutes. Organoids were dissociated and cells seeded onto the Matrigel-coated wells in 5% organoid media supplemented with 10% FBS. Monolayer cultures were grown for 72 hours and RNA was extracted for qRT-PCR.

To evaluate organoid-forming potential, 2,000 organoid-derived cells were plated in 10 μL of Matrigel and allowed to solidify. Cultures were grown in organoid media for 3 days, and the number of organoids within each well was counted. To monitor organoid growth over time, 2,000 cells were seeded into organoid culture, and at days 3, 6, and 9, cultures were harvested and dissociated into single-cell suspensions using trypsin. Live cells (trypan blue excluding) were counted using a hemocytometer (Thermo Fisher Scientific). At least 3 biological replicates (i.e., 3 distinct organoid cultures) were analyzed.

KC organoids with ST6GAL1 knockdown (KC-KD) were generated by transducing dissociated organoid cells with lentivirus encoding shRNA against *St6gal1*, using the vector described above for 266-6 cells. Control organoids (KC-shC) were generated using lentivirus encoding a nontargeting shRNA (MilliporeSigma SHC005V). Approximately 50,000 organoid cells were mixed with lentivirus at a MOI of 5 and incubated at 37°C for 6 hours. After washing to remove lentiviral particles, cells were resuspended in Matrigel and plated to allow reformation of the organoids. Stably transduced organoids were selected with puromycin.

### RNA extraction and qRT-PCR

RNA was extracted using the Ambion RNA extraction kit (Thermo Fisher Scientific). The M-MLV reverse transcriptase (Promega) was used for cDNA preparation. qRT-PCR was conducted using the TaqMan Fast Advanced Master Mix (Thermo Fisher Scientific) using primers for *Sox9* (Mm00448840_m1), *Hes1* (Mm01342805_m1), *Ptf1a* (Mm00479622_m1), *Lgr5* (Mm000438890_m1), and *Axin2* (Mm00443610_m1). *Gapdh* primers (Mm99999915_g1) were used for normalization. At least 3 independent experiments were performed, with each experiment performed in triplicate.

### Immunoblotting

Cell lines were lysed in RIPA buffer containing protease and phosphatase inhibitors (Thermo Fisher Scientific). For organoid lysis, organoids were scraped into PBS-EDTA and disrupted manually to break up organoid-Matrigel clumps. Samples were centrifuged at 300*g* for 5 minutes at 4ºC and incubated in trypsin to digest the Matrigel. Dissociated organoid cells were lysed in RIPA buffer with protease and phosphatase inhibitors. Lysate protein concentrations were quantified by BCA (Thermo Fisher Scientific). Following SDS-PAGE, proteins were transferred to PVDF membranes. Membranes were blocked in 5% nonfat dry milk and incubated overnight at 4°C with antibodies against SOX9, HES1, PTF1A, EGFR, p-EGFR (pTyr-1068), and ST6GAL1 (see Supplemental Table 3). Membranes were then incubated in secondary antibody and developed using Clarity Western ECL substrate (Bio-Rad). Densitometry was conducted using ImageJ (NIH) on blots from at least 3 independently generated lysates.

### Cerulein treatment and flow cytometric analysis of ADM cells

To induce pancreatitis, mice were treated with 7 intraperitoneal injections of cerulein (50 μg/kg; Tocris Bioscience), or saline control, at hourly intervals on 2 alternating days as described before (52). On day 4, pancreata were digested for 30 minutes using the Tissue Dissociation Kit (Miltenyi Biotec) supplemented with a ROCK inhibitor. Digested tissue was passed through a 100 μm filter and centrifuged at 300*g* for 5 minutes at 4°C. The pellet was resuspended in PBS with ROCK inhibitor. Under these conditions, the viability of dissociated cells was more than 90%. The cell suspension was stained with Aqua live/dead stain, anti-EpCAM–PE, anti-CD45–APC, anti-CD133–PE/Cy-7, and UEA-FITC for 30 minutes (see Supplemental Table 3). UEA and anti-CD133 staining was quantified on cells positive for EpCAM (epithelial marker), negative for CD45 (immune marker), and negative for the Aqua dye. Unstained and single-color controls were used to set voltage and compensation, and only viable singlets were counted (*n* = 4 mice/group).

### Serum amylase measurements

Pancreatitis was induced by cerulein injections as above. At 12 hours following the last injection, blood was collected from the caudal vena cava into BD Microtainer tubes (Thermo Fisher Scientific). Samples were centrifuged at 2,000*g* for 5 minutes at 4°C to obtain serum. An Amylase Assay Kit (Abcam) was used to determine serum amylase levels.

### Histological analyses

#### IHC.

Paraffin-embedded tissues were processed for antigen retrieval and IHC-stained for ST6GAL1 and/or SOX9 as described before (26, 39). Slides were counterstained with hematoxylin (Vector Labs), dehydrated, and mounted with coverslips using VectaMount Medium (Vector Labs).

#### Immunofluorescence staining.

Paraffin-embedded tissues were incubated overnight at 4°C with antibodies against SOX9, EGFR, p-EGFR (pTyr-1068), GM130, pancreatic α amylase, KRT8, KRT19, and ST6GAL1 (see Supplemental Table 3). Slides were then incubated with secondary antibody, followed by incubation in Hoechst solution for 5 minutes. Slides were mounted using VECTASHIELD Vibrance Antifade Mounting Medium (Vector Labs).

#### H&E, Alcian blue, and Sirius red staining.

For H&E staining, slides were exposed to hematoxylin for 7 minutes (VitroVivo), then immersed in 95% alcohol for 1 minute, followed by eosin staining for 30 seconds. H&E-stained slides were analyzed for PanINs and adenocarcinoma under masked conditions by a board-certified pathologist. Tissues were also stained with (i) Alcian blue (IHC World) followed by counterstaining with Nuclear Fast Red (Vector Labs) and (ii) Sirius red (IHC World) followed by counterstaining with hematoxylin. Staining was quantified using a stereological approach. A grid was placed over an image of the entire pancreas, and the intersection points landing on tissues positively stained for either Alcian blue or Sirius red were counted. To obtain the stereologic score, the number of intersection points landing on positively stained tissues was divided by the total number of intersection points counted for the slide.

### RNA-Seq

#### RNA isolation and sequencing.

Pancreata were placed in RNAlater (Invitrogen) overnight at 4°C. Samples were homogenized in TRIzol (Thermo Fisher Scientific), and 100 μL chloroform was added for each milliliter of TRIzol. The top aqueous layer was collected, and an equal volume of 70% ethanol was added. RNA was isolated using RNeasy Mini Kit (QIAGEN). For organoids, cells were first dissociated, and RNA was extracted as above. RNA-Seq was conducted using Illumina’s NextSeq550 and NovaSeq 6000 at the UAB Genomics Core. RNA quality was assessed using the Agilent 2100 Bioanalyzer, and RNA with an RNA integrity number ≥ 7.0 was used for library preparation. RNA was converted to a sequencing-ready library using the NEBNext Ultra II Directional RNA library kit (New England Biolabs). The cDNA libraries were quantitated using quantitative PCR in a Roche LightCycler 480 with the Kapa Biosystems kit for Illumina library quantitation.

#### RNA-Seq analysis.

STAR (version 2.7.10a) was used to align the fastq sequences to Gencode’s mouse genome (GRCm39 Release M32) (82). HTSeq-count (version 2.0.2) was used to estimate transcript abundances (83). DESeq2 (version 1.38.3) normalized and calculated differential expression following their vignette (84).

#### IPA.

A data set containing gene identifiers and corresponding expression values was uploaded into IPA (85). A fold-change cutoff of ±2 and *P* < 0.05 was set to define network-eligible molecules.

#### GSEA.

GSEA (version 4.1.0) was performed on the normalized gene expression data using the Molecular Signatures Database (version 7.2) (86, 87). Default parameters were used, except the permute parameter was set to gene set, and plot graphs for the top sets of each phenotype were set to 50.

### Statistics

Statistical analyses were conducted using GraphPad Prism (version 9.5.1). ST6GAL1 expression in human pancreatic specimens was analyzed using the nonparametric Kruskal-Wallis test followed by Dunn’s multiple-comparison test. A nonparametric test was selected after evaluating the data with the D’Agostino-Pearson normality test. For the tumor xenograft studies, data were analyzed using a 2-way ANOVA or Mann-Whitney *U* test. Median survival time for KC and KSC mice was calculated using a Kaplan-Meier analysis. For other experiments, data were evaluated using a 2-tailed Student’s *t* test, 1-way ANOVA, or 2-way ANOVA, where *P* < 0.05 was considered significant. Graphs depict mean ± SEM.

### Study approval

All animal studies were approved by the UAB Institutional Animal Care and Use Committee. Both males and females were included.

### Data availability

All data are contained within the manuscript or supplemental materials, with the exception of the RNA-Seq results, which have been deposited in NCBI’s Gene Expression Omnibus. RNA-Seq data are available through accession numbers GSE169546 (pancreatic tissues) and GSE234045 (organoids). Values for all data points on graphs are reported in the Supporting Data Values file.

## Author contributions

NB, AC, MPM, JH, CMB, ADS, RBJ, DKC, and MRC contributed to data curation. AC, NB, MPM, CMB, DKC, IEE, and SLB were involved in formal analysis of the data. CAK and DJB provided resources, and AC, NB, MPM, CMB, KLA, LES, PDS, BS, LEH, MMB, CSH, and ZY assisted in developing the methodology. Composition of the original draft was executed by AC, NB, and SLB, and the manuscript was edited and reviewed by all authors. NB and AC are shared first authors, and NB’s name appears first based on alphabetical ordering. SLB was responsible for project administration and funding acquisition.

## Supplementary Material

Supplemental data

Supporting data values

## Version 1 08/29/2023

In-Press Preview

## Version 2 10/09/2023

Electronic publication
