# Supplementary material for: ST6GAL1 sialyltransferase promotes acinar to ductal metaplasia and pancreatic cancer progression
Source: JCI Insight. 2023 Oct 9;8(19):e161563. doi: 10.1172/jci.insight.161563 (PMC10619436; doi:10.1172/jci.insight.161563)
Supplement: Supplemental data [file jciinsight-8-161563-s009.pdf]

SUPPLEMENTARY MATERIALS

ST6GAL1 sialyltransferase promotes acinar to ductal metaplasia and pancreatic cancer progression

Nikita Bhalerao[1#], Asmi Chakraborty[1#], Michael P. Marciel[1], Jihye Hwang[1], Colleen M. Britain[1], Austin D. Silva[1], Isam E. Eltoum[2], Robert B. Jones[1], Katie L. Alexander[3], Lesley E. Smythies[3], Phillip D. Smith[3], David K. Crossman[4], Michael R. Crowley[4], Boyoung Shin[1], Laurie E. Harrington[1], Zhaoqi Yan[1], Maigen M. Bethea[3], Chad S. Hunter[3,] Christopher A. Klug[5], Donald J. Buchsbaum[6], and Susan L. Bellis[1*]

To whom correspondence should be addressed:

Susan L. Bellis, Ph.D.
University of Alabama at Birmingham
bellis@uab.edu

Supplementary Materials include:

Supplementary Figure 1: Representative images of ST6GAL1 expression in human PDAC patient tissues.
Supplementary Figure 2: SNA staining of surface α2,6 sialic acids in the Suit2 isogenic cell series. Also included are imaging and histological analyses conducted on Suit2, S2-LM7AA and S2-013-derived primary tumors and metastases.
Supplementary Figure 3: H&E and ST6GAL1 IHC staining of tissues from pancreas and other organs in WT and SC mice.
Supplementary Figure 4: H&E and ST6GAL1 IHC staining of tissues from pancreas and other organs in KC and KSC mice.
Supplementary Figure 5: SOX9 staining of acinar and ductal cells from GEM tissues.
Supplementary Table 1: Extended GSEA analyses of SC and WT pancreata.
Supplementary Table 2: Extended GSEA analyses of KSC and KC pancreata.
Supplementary Table 3: Antibody and lectin information.

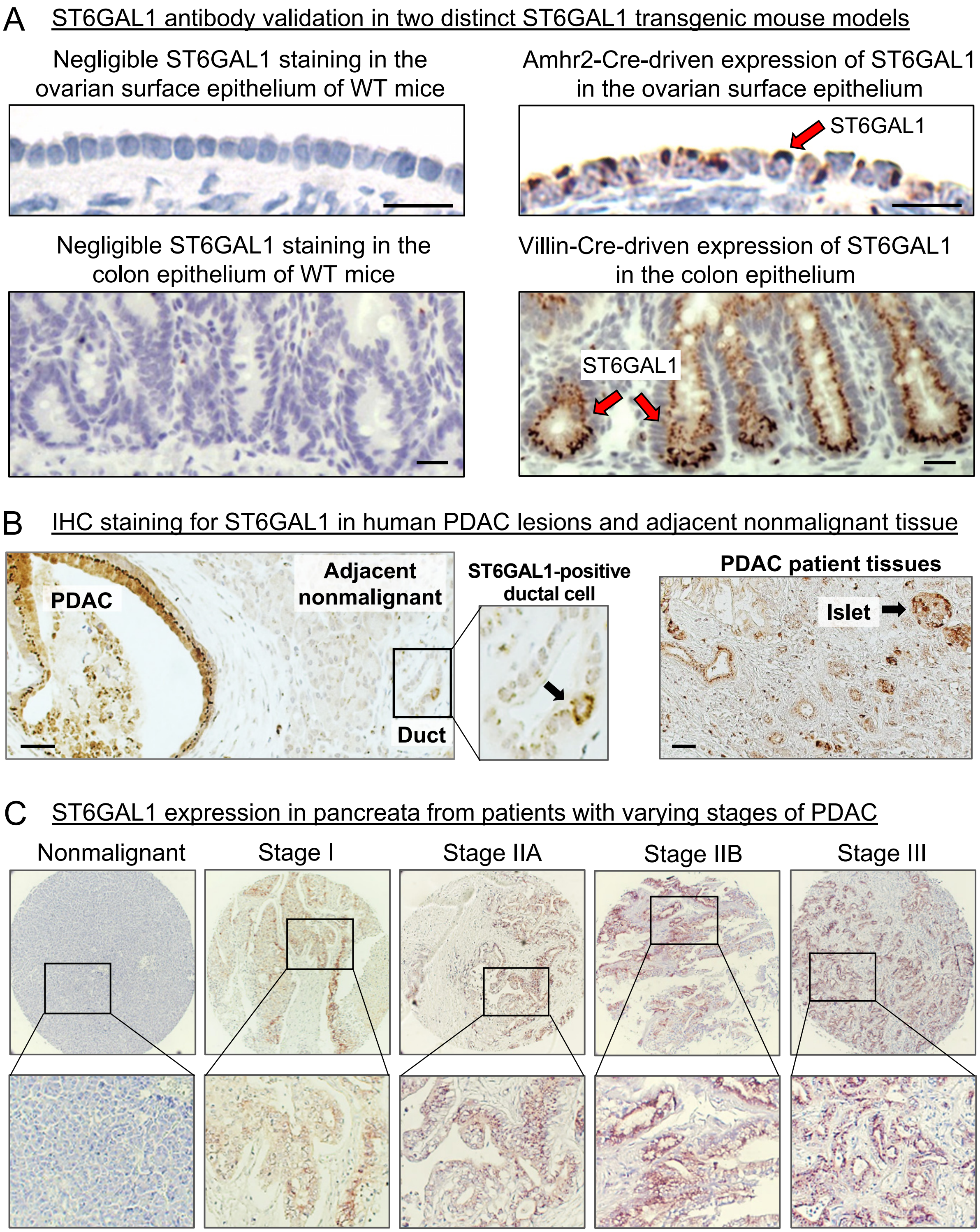

Supplementary Figure 1. ST6GAL1 expression in nonmalignant and malignant patient pancreatic tissues

(A) The ST6GAL1 antibody was validated by IHC staining in two GEM models with ectopic expression of ST6GAL1 (note that this antibody recognizes both human and murine ST6GAL1). Strong ST6GAL1 staining was noted in the ovarian epithelium of mice with Amhr2-Cre driven ST6GAL1 expression (top right panel), as well as in colon epithelium of mice with Villin-Cre driven ST6GAL1 expression (lower right panel). Scale bar = 20 μM.

(B) IHC staining for ST6GAL1 in a PDAC patient sample. ST6GAL1 is extensively expressed throughout the PDAC lesion, whereas expression is negligible in the nonmalignant, adjacent pancreatic tissue. The inset depicts a rare, ST6GAL1-positive ductal cell within the nonmalignant adjacent tissue. ST6GAL1 is also expressed in islet cells (right panel). Scale bar = 50 μM.

(C) ST6GAL1 IHC staining in representative specimens from patients with varying stages of PDAC.

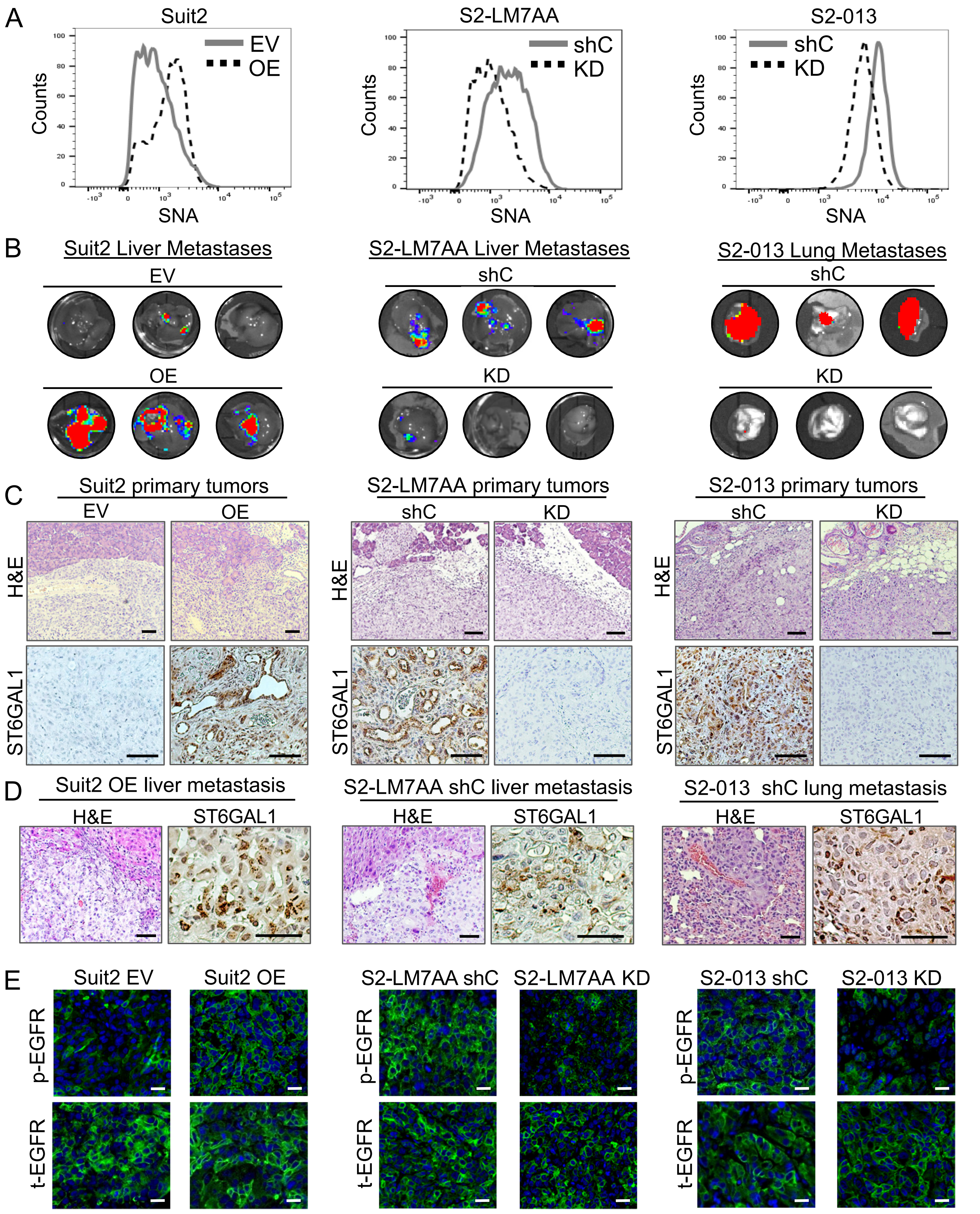

Supplementary Figure 2. Tumor xenografts using the isogenic human Suit2 PDAC cell series.
(A) Cells with modulated ST6GAL1 expression (overexpression, OE, or knockdown, KD) were stained with the SNA lectin, which binds α2,6 sialic acids, and analyzed for surface sialylation by flow cytometry.

Supplementary Figure 2. Tumor xenografts using the isogenic human Suit2 PDAC cell series (continued).

(B) Bioluminescence imaging (BLI) of representative organs harboring metastatic tumors formed from Suit2, S2-LM7AA and S2-013 cells. Each image is from a distinct mouse.

(C) Upper panels: H&E stained primary tumors from the Suit2, S2-LM7AA and S2-013 cohorts. Lower panels: IHC staining for ST6GAL1 on primary tumors from the Suit2, S2-LM7AA and S2-013 cohorts. Suit2 and S2-LM7AA cells were injected into the pancreas; S2-013 cells were injected into the flank. Scale bar = 100 μM

(D) H&E (left panels) or IHC staining for ST6GAL1 (right panels) on metastatic tumors from the cell lines with high ST6GAL1 expression (Suit2 OE; S2-LM7AA shC; S2-013 shC). Scale bar = 50 μM

(E) Staining for p-EGFR and t-EGFR in primary xenograft tumors extracted from mice injected with Suit2, S2-LM7AA and S2-013 cells. Scale bar = 25 μM

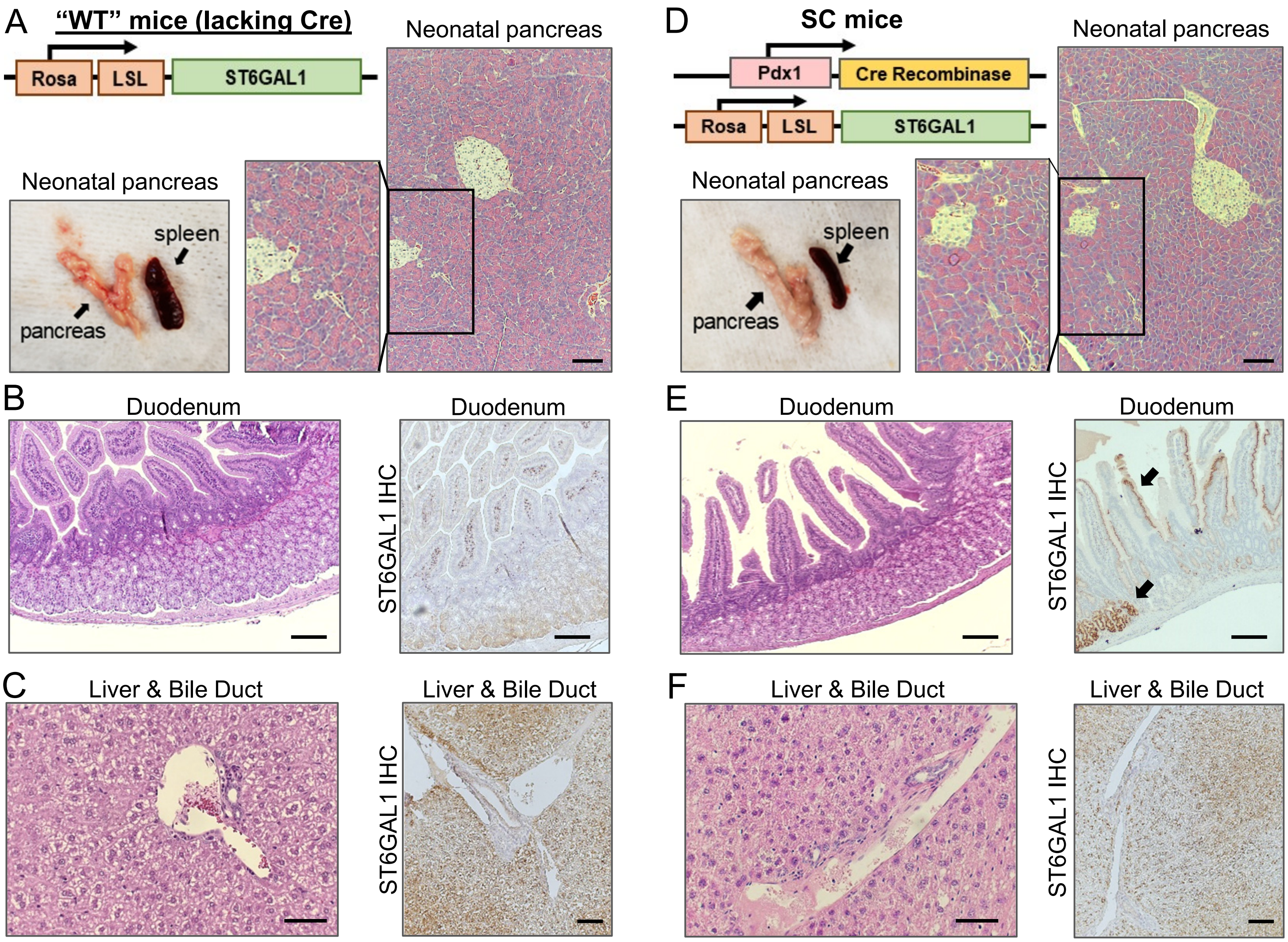

Supplementary Figure 3. Histologic evaluation of tissues from WT and SC GEM models

(A) Image of whole pancreas and H&E stained pancreatic tissues from neonatal mice expressing the *ST6GAL1* transgene, but not the Cre recombinase, abbreviated as "WT" mice. Scale bar = 100 μM.

(B) H&E staining and ST6GAL1 IHC on duodenal tissues from WT mice. Notably, some stromal cells stain positively for endogenous ST6GAL1, consistent with the well-known expression of ST6GAL1 in certain immune cell populations. Scale bars = 50 μM.

(C) H&E staining and ST6GAL1 IHC on liver and bile duct of WT mice. Hepatocytes are known to express ST6GAL1. Scale bars = 25 μM.

(D) Image of pancreas and H&E stained tissues from neonatal SC mice (expressing the *ST6GAL1* transgene plus Cre recombinase). No abnormalities were detected in neonatal SC pancreata. Scale bar = 100 μM.

(E) H&E staining and ST6GAL1 IHC on duodenal tissues from SC mice. Some expression of the *ST6GAL1* transgene is detected in the duodenum (arrows). Pdx1-Cre is known to drive recombination in the duodenum. Scale bars = 50 μM.

(F) H&E staining and ST6GAL1 IHC on liver and bile duct of SC mice. Scale bars = 25 μM.

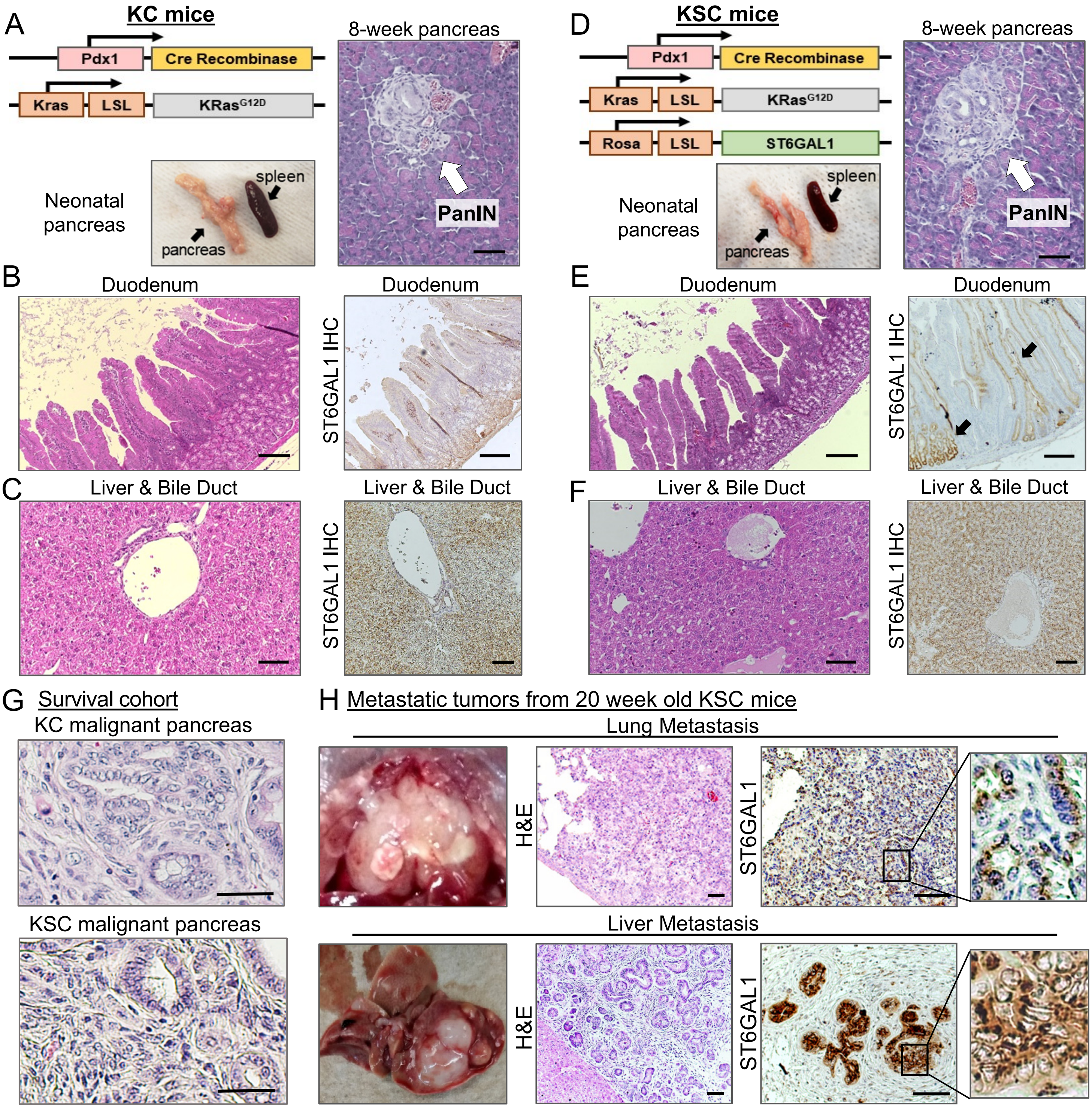

Supplementary Figure 4. Histologic evaluation of tissues from KC and KSC mice

(A) Image of pancreas and H&E stained pancreatic tissues from KC mice. No abnormalities were noted in neonatal mice, however, PanIN lesions were detected in 8-week old KC mice. Scale bar = 50 μM.

(B) H&E and ST6GAL1 IHC on duodenal tissues from KC mice. Scale bars = 50 μM.

(C) H&E and ST6GAL1 IHC on liver and bile duct from KC mice. Scale bars = 25 μM.

(D) Image of pancreas and H&E stained tissues from KSC mice. No pancreatic abnormalities were observed in neonatal mice, however PanIN lesions were apparent in 8-week old mice. Scale bar = 50 μM.

(E) H&E and ST6GAL1 IHC on duodenal tissues from KSC mice. Some expression of the *ST6GAL1* transgene is observed in the duodenum (arrows). Scale bars for H&E and IHC = 50 μM.

(F) H&E and ST6GAL1 IHC on liver and bile duct from KSC mice. Scale bars = 25 μM.

(G) H&E stained pancreata from KC and KSC mice in the survival cohort. Scale bars = 50 μM.

(H) Metastatic tumors from 20 week old KSC mice. Upper panels depict lung metastases. Lower panels depict liver metastases: Scale bars = 100 μM.

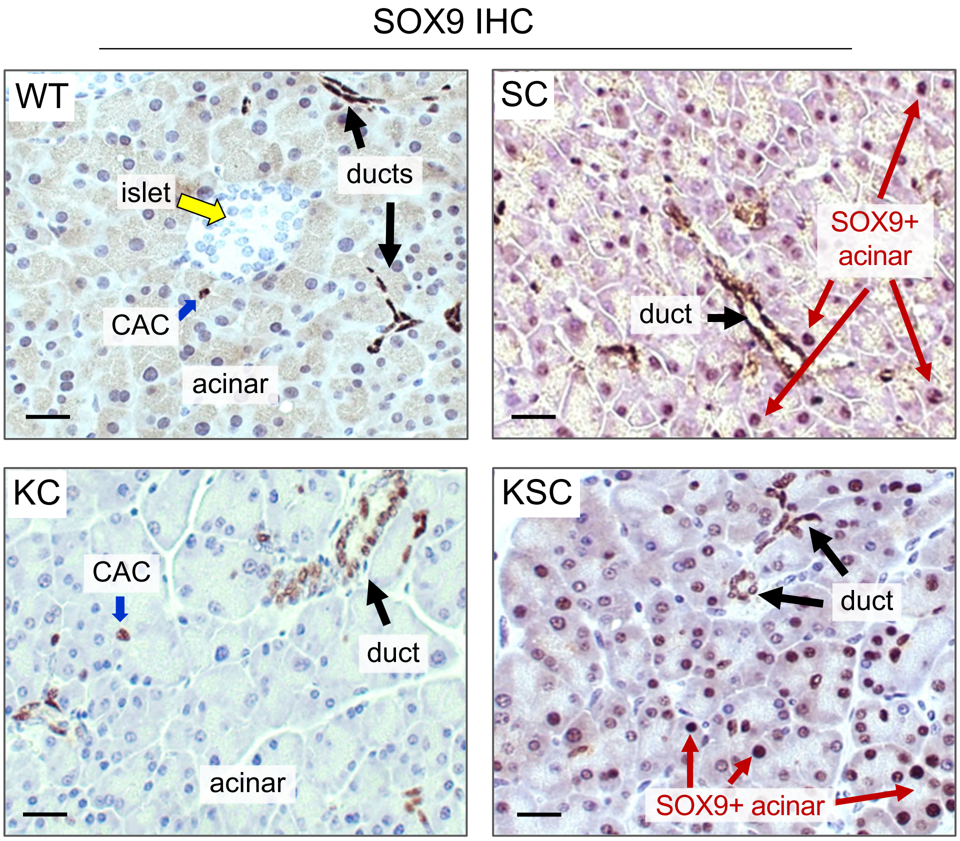

Supplementary Figure 5. Expression of SOX9 in acinar and ductal cells

SOX9 IHC was conducted on pancreatic tissues from WT, SC, KC and KSC mice. In WT mice, SOX9 is expressed in normal ductal cells (black arrows) and some centroacinar cells (CAC), but not in islet cells (yellow arrows) or mature acinar cells. In contrast to WT and KC pancreata, mature acinar cells from SC and KSC mice abnormally express SOX9 (red arrows). Scale bars = 25 µM

Supplementary Table 1. GSEA of pathways upregulated in SC mice vs. WT mice

PATHWAYS	NES	FDR
Developmental/Stemness Pathways		
GO EXOCRINE SYSTEM DEVELOPMENT	2.33	0.001
GO CELL MORPHOGENESIS	2.21	0.002
HALLMARK WNT BETA CATENIN SIGNALING	1.59	0.018
GO BETA CATENIN TCF COMPLEX ASSEMBLY	2.10	0.006
PID HES HEY PATHWAY	1.57	0.089
REACTOME REGULATION OF RUNX1 EXPRESSION AND ACTIVITY	2.01	0.015
PID BMP PATHWAY	1.82	0.021
WP HIPPOYAP SIGNALING PATHWAY	1.59	0.086
Growth Factor Receptor Signaling Pathways		
GO TRANSMEMBRANE PROTEIN RECEPTOR KINASE ACTIVITY	2.15	0.004
REACTOME SIGNALING BY RECEPTOR TYROSINE KINASES	1.74	0.050
WP EGFR TYROSINE KINASE INHIBITOR RESISTANCE	1.83	0.027
REACTOME SIGNALING BY ERBB2 IN CANCER	1.88	0.027
REACTOME MET PROMOTES CELL MOTILITY	2.08	0.009
REACTOME SIGNALING BY FGFR1 IN DISEASE	2.21	0.004
PID VEGFR1_2 PATHWAY	1.83	0.021
REACTOME SIGNALING BY PDGF	2.16	0.004
Signaling by Small G Proteins		
GO SMALL GTPASE BINDING	2.43	0.001
GO RAS GUANYL NUCLEOTIDE EXCHANGE FACTOR ACTIVITY	2.24	0.002
PID RHOA REG PATHWAY	2.00	0.009
GO RAB GTPASE BINDING	2.44	0.001
Cell Adhesion and Integrin Signaling		
PID N-CADHERIN PATHWAY	1.96	0.016
PID INTEGRIN1 PATHWAY	1.88	0.020
PID AVB3 INTEGRIN PATHWAY	1.84	0.020
PID FAK PATHWAY	1.92	0.019
KEGG FOCAL ADHESION	2.06	0.006
Cancer Pathways		
KEGG PATHWAYS IN CANCER	1.78	0.020
WP BREAST CANCER	1.93	0.017
WP BLADDER CANCER	1.74	0.048
KEGG RENAL CELL CARCINOMA	1.68	0.030
KEGG PROSTATE CANCER	1.63	0.045
KEGG THYROID CANCER	1.82	0.015
KEGG NON SMALL CELL LUNG CANCER	1.76	0.020
KEGG ENDOMETRIAL CANCER	1.73	0.026
KEGG GLIOMA	1.76	0.020
KEGG CHRONIC MYELOID LEUKEMIA	1.69	0.030

Supplementary Table 2. GSEA of pathways upregulated in KSC mice vs. KC mice

PATHWAYS	NES	FDR
Developmental/Stemness Pathways		
GO REGULATION OF EMBRYONIC DEVELOPMENT	2.090	0.014
REACTOME TRANSCRIPTIONAL REGULATION OF PLURIPOTENT STEM CELLS	1.875	0.047
GO POSITIVE REGULATION OF STEM CELL PROLIFERATION	2.223	0.011
GO CELL MORPHOGENESIS	2.036	0.014
REACTOME REGULATION OF RUNX1 EXPRESSION AND ACTIVITY	2.060	0.011
PID KIT PATHWAY	1.649	0.045
PID BMP PATHWAY	1.853	0.024
PID HES HEY PATHWAY	1.616	0.052
GO HIPPO SIGNALING	2.101	0.016
HALLMARK EPITHELIAL MESENCHYMAL TRANSITION	1.727	0.005
Growth Factor Receptor Signaling Pathways		
GO TRANSMEMBRANE RECEPTOR PROTEIN TYROSINE KINASE ACTIVITY	1.852	0.031
BIOCARTA EGF PATHWAY	1.939	0.024
GO EPIDERMAL GROWTH FACTOR RECEPTOR BINDING	1.710	0.060
BIOCARTA HER2 PATHWAY	2.029	0.045
BIOCARTA MET PATHWAY	1.656	0.076
REACTOME MET PROMOTES CELL MOTILITY	1.942	0.026
WP PDGF PATHWAY	1.697	0.048
WP PDGFRBETA PATHWAY	1.959	0.016
KEGG TGFBETA SIGNALLING PATHWAY	1.647	0.082
Signaling by Small G Proteins		
HALLMARK KRAS SIGNALING UP	1.877	0.002
PID RHOA REG PATHWAY	1.665	0.042
BIOCARTA RAC1 PATHWAY	1.660	0.079
Cell Adhesion and Integrin Signaling		
PID N-CADHERIN PATHWAY	1.715	0.034
PID INTEGRIN1 PATHWAY	1.805	0.033
PID AVB3 INTEGRIN PATHWAY	1.799	0.028
GO FOCAL ADHESION ASSEMBLY	2.074	0.010
PID FAK PATHWAY	1.862	0.024
GO FILOPODIUM	1.595	0.079
GO LAMELLAPODIUM	1.901	0.043
GO INVADOPODIUM	1.768	0.035
Cancer Pathways		
WP GASTRIC CANCER NETWORK	1.755	0.038
WP HEAD AND NECK SQUAMOUS CELL CARCINOMA	1.801	0.031
KEGG SMALL CELL LUNG CANCER	1.670	0.083
KEGG BASAL CELL CARCINOMA	1.582	0.097

Supplementary Table S3: Antibody and Lectin information

Antibody or Lectin	Application	Dilution	Company (Catalogue #)
ST6GAL1	IHC, IF	1:75	R&D systems (AF5924)
Sox9	IHC, IF	1:250	Abcam (ab185230)
EGFR	IF	1:250	Abcam (ab52894)
p-EGFR (Tyr1068)	IF	1:250	Abcam (ab40815)
GM130	IF	1:200	Abcam (ab52649)
Pancreatic α amylase	IF	1:2000	Abcam (ab199132)
KRT8	IF	1:100	Thermo Fisher (PA5-29607)
KRT19	IF	1:100	Proteintech (107-12-1AP)
EpCAM-PE	Flow cytometry	1:200	BioLegend (118205)
Aqua Live-Dead stain	Flow cytometry	1:1000	ThermoFisher (L34957)
SNA-FITC	Flow cytometry	1:200	Vector laboratories (FL-1301-2)
CD45-APC	Flow cytometry	1:200	BioLegend (103111)
CD133-PE/Cy7	Flow cytometry	1:200	BioLegend (141209)
UEA-FITC	Flow cytometry	1:200	Sigma (L9006)
ST6GAL1	WB	1: 500	R&D systems (AF5924)
Sox9	WB	1:1000	Abcam (ab185230)
Hes1	WB	1:1000	Abcam (ab71559)
Ptf1a	WB	1:2000	Abcam (ab182398)
EGFR	WB	1:1000	CST (4267)
p-EGFR (Tyr1068)	WB	1:1000	CST (3777)
